# Membrane-decisional architecture: a framework for multigenomic signal prioritization in the plasma membrane nanobiome

**DOI:** 10.3389/fphys.2026.1828577

**Published:** 2026-08-18

**Authors:** João Francisco Pollo Gaspary, Luis Felipe Dias Lopes, Fernanda Peron Gaspary, Carmen Brum Rosa, Eduarda Grando Lopes, Alfred Lee Edgar, Eduardo Poletti Camara, Antonio Geraldo Camara

**Affiliations:** 1 Institute AuBento – Center for Education, Clinical Practice, and Research in Orthomolecular and Integrative Medicine, Santa Maria, Brazil; 2 Center for Social and Human Sciences, Postgraduate Program in Administration and Accounting, Federal University of Santa Maria, Santa Maria, Brazil; 3 Area of Technological Sciences, Franciscan University, Santa Maria, Brazil; 4 Production Engineering Department, Federal University of Santa Maria, Santa Maria, Brazil; 5 Veterinary Medicine Course, Federal University of Santa Maria, Santa Maria, Brazil; 6 Research and Development Department, ElastroCrete, LLC, Veyo, UT, United States; 7 Institute Camara – Center for Clinical and Orthomolecular Practice, Ribeirão Preto, Brazil; 8 Department of Cardiology and Internal Medicine, Beneficência Portuguesa de São Paulo, Ribeirão Preto, Brazil

**Keywords:** endocytic routing, membrane microdomains, membrane physiology, membrane-decisional architecture, receptor clustering, signaling competence, systems physiology, multigenomic physiology

## Abstract

**Background:**

Classical models of cellular signaling assume that ligand recognition at the plasma membrane is sufficient to initiate intracellular cascades. However, experimental evidence consistently shows that extracellular signals can engage receptors without producing functional cellular responses and that identical ligand–receptor interactions may generate divergent outcomes depending on membrane organization, receptor clustering, and intracellular routing dynamics.

**Conceptual framework:**

To address this discrepancy, the concept of membrane**-**decisional architecture is introduced, in which plasma membrane organization is interpreted as a signal prioritization interface. Within this framework, membrane structure governs signaling competence by regulating signal access, receptor clustering, intracellular routing, and compartmental signal conversion, thereby determining which extracellular inputs acquire functional relevance.

**Methods:**

A structured integrative synthesis was conducted using a Work Breakdown Structure (WBS) framework (WP1–WP5), encompassing evidence acquisition, mechanistic mapping, integrative synthesis, cross**-**system structural calibration, and formal systems integration. Mechanistic findings from membrane biology and cellular signaling were reorganized to evaluate whether a coherent architecture of signaling competence emerges across biological systems. A conceptual mathematical formalization was developed to represent the relationship between signal availability and membrane**-**dependent competence.

**Implications:**

This framework does not propose new molecular mechanisms but provides an integrative interpretation of how established membrane**-**level processes collectively constrain signaling outcomes under heterogeneous input conditions. Membrane organization is therefore positioned as an upstream determinant of cellular responsiveness. The proposed formalization provides a basis for experimental investigation of membrane**-**dependent signaling competence and its role in physiological variability.

## 1 Introduction

Cellular signaling is traditionally described as a receptor**-**driven process in which ligand recognition at the plasma membrane initiates intracellular signaling cascades leading to transcriptional responses and physiological outcomes (Medzhitov, 2008). Within this framework, ligand presence and receptor interaction are assumed to be the primary determinants of signaling activation. Advances in molecular and structural biology have refined this view, establishing detailed models of receptor–ligand interactions and downstream pathways across diverse physiological systems (Lingwood and Simons, 2010; Chen et al., 2023; Armstrong et al., 2025).

However, experimental observations across multiple domains reveal a persistent discrepancy. Extracellular signals may be present and capable of engaging receptors without producing functional cellular responses. Identical ligands interacting with the same receptors can generate distinct outcomes depending on membrane organization, receptor clustering, and intracellular trafficking (Lingwood and Simons, 2010; Changede and Sheetz, 2017; Shi et al., 2018; Armstrong et al., 2025). Similar patterns have been reported in innate immune signaling, viral nucleic acid sensing, growth factor receptor activation, and metabolic pathways (Sun et al., 2009; Watanabe et al., 2011; Kleinman et al., 2012; Koarai et al., 2012; Fork et al., 2014). These findings indicate that ligand recognition alone does not fully explain the transition from signal presence to signaling competence.

Plasma membrane organization plays a central role in shaping these outcomes (Lingwood and Simons, 2010; Sahl et al., 2017; Armstrong et al., 2025). Cholesterol**-** and sphingolipid**-**enriched microdomains act as spatial organizers of receptor complexes and signaling assemblies (Li et al., 2004; Lingwood and Simons, 2010). Within these domains, receptors, adaptor proteins, enzymes, and cytoskeletal elements assemble into dynamic platforms that modulate signal amplitude and persistence (Yin and Stull, 1999; Heikkinen et al., 2009; Changede and Sheetz, 2017; Guerra et al., 2022).

Despite these advances, the role of membrane organization remains incompletely integrated into physiological interpretations of signal regulation (Medzhitov, 2008; Netea et al., 2016; Netea et al., 2020). Rather than acting only as facilitators of receptor interaction, membrane structures may function as upstream constraints that determine whether signals acquire biological competence. Clustering (Armstrong et al., 2025), adaptor recruitment (Chen et al., 2023), and trafficking pathways (Watanabe et al., 2011; Fork et al., 2014) influence whether signals are internalized, amplified, attenuated, or rendered functionally silent. This suggests that membrane organization determines which signals become biologically effective.

This becomes particularly relevant in heterogeneous extracellular environments. Human cells are continuously exposed to signals from host processes, microbiota**-**derived metabolites, viral nucleic acids, and environmental factors (Douglas, 2010; Human Microbiome Project Consortium, 2012; McFall-Ngai et al., 2013; Liang and Bushman, 2021). Recent system-level analyses further support this interpretation by demonstrating that the human organism operates as a structurally asymmetric multigenomic system, in which host and exogenous biological networks continuously interact through shared physiological interfaces (Gaspary et al., 2026a). These inputs converge at the plasma membrane, where they interact within a shared interface. Under such conditions, signaling outcomes cannot be interpreted solely as isolated receptor–ligand events but as structured integration under coexistence and competition among heterogeneous inputs (Netea et al., 2016; Netea et al., 2020).

The present study examines whether membrane organization may provide the substrate through which this integration is resolved. Within the conceptual framework developed here, this substrate is interpreted as the plasma membrane nanobiome—a membrane-centered interface that provides the organizational context for multigenomic signal prioritization. Importantly, this work is presented as a hypothesis-derived conceptual framework rather than a validated model. The concept of membrane-decisional architecture is proposed to describe the plasma membrane as a signal prioritization interface under multigenomic input conditions. Within this membrane-centered organizational framework, signaling competence is not determined solely by ligand presence but by membrane-dependent conditions regulating signal accessibility, receptor clustering, and intracellular routing, offering a structured interpretation of why identical stimuli produce divergent outcomes.

Methodologically, the framework is developed through structured conceptual synthesis, including evidence acquisition, mechanistic mapping, integrative synthesis, cross**-**system calibration, and system**-**level integration. Established findings from membrane biology and signaling research are reorganized to evaluate whether a coherent architecture of signaling competence emerges across biological systems.

Within this perspective, the transition from signal presence to signaling competence is interpreted as a structured process governed by membrane**-**dependent conditions. This relationship can be conceptualized as a constraint**-**based interaction between signal availability and membrane competence, providing the basis for system**-**level representations of signaling behavior.

To further articulate this interpretation, the plasma membrane nanobiome is introduced as a unifying description of the membrane interface. The nanobiome is defined as the nanoscale physicochemical environment in which membrane organization, molecular interactions, and microenvironmental conditions converge, including lipid structure, protein complexes, the glycocalyx, and adjacent intra**-** and extracellular spaces. Within this interface, proton dynamics, redox balance, surface charge, and structural organization define the conditions under which signaling competence emerges.

Rather than introducing new mechanisms, the nanobiome provides an integrative interpretation of how established determinants shape membrane**-**dependent signaling. It organizes an already observed but underintegrated phenomenon: identical extracellular inputs may produce divergent cellular outcomes depending on membrane**-**associated conditions.

Finally, the term “multigenomic” is used in an extended sense, encompassing not only signals encoded by distinct genomic systems but also the physiological field emerging from their interaction. Metabolic states, redox balance, extracellular pH, and gaseous environments are therefore interpreted as downstream expressions of multigenomic interactions, forming a coupled regulatory landscape of genomic and nongenomic variables. This interpretation is further supported by the recently published structural model of the multigenomic human system, in which host and exogenous biological processes are understood as components of a continuously interacting physiological network (Gaspary et al., 2026a).

## 2 Methods

The present study employed a structured conceptual synthesis guided by a Work Breakdown Structure (WBS)-based methodological framework (Project Management Institute, 2019; Project Management Institute, 2021) to examine how plasma membrane organization influences the emergence of signaling competence in human cellular systems. Rather than focusing on clinical outcomes or intervention effects, the objective was to integrate experimentally demonstrated mechanisms from membrane biology and cell signaling to clarify how extracellular signals acquire functional regulatory relevance at the cellular interface (Kitano, 2002).

Given the heterogeneity of the underlying experimental domains—including membrane biophysics, receptor trafficking, innate immune sensing, and intracellular signaling—traditional systematic review approaches based on effect-size aggregation were not suitable. Instead, experimentally demonstrated mechanisms were examined for structural convergence and progressively reorganized into a coherent conceptual architecture through sequential analytical stages (WP1–WP5), encompassing evidence acquisition, mechanistic mapping, cross-domain integration, plausibility assessment, and framework formalization.

**Figure 1:**
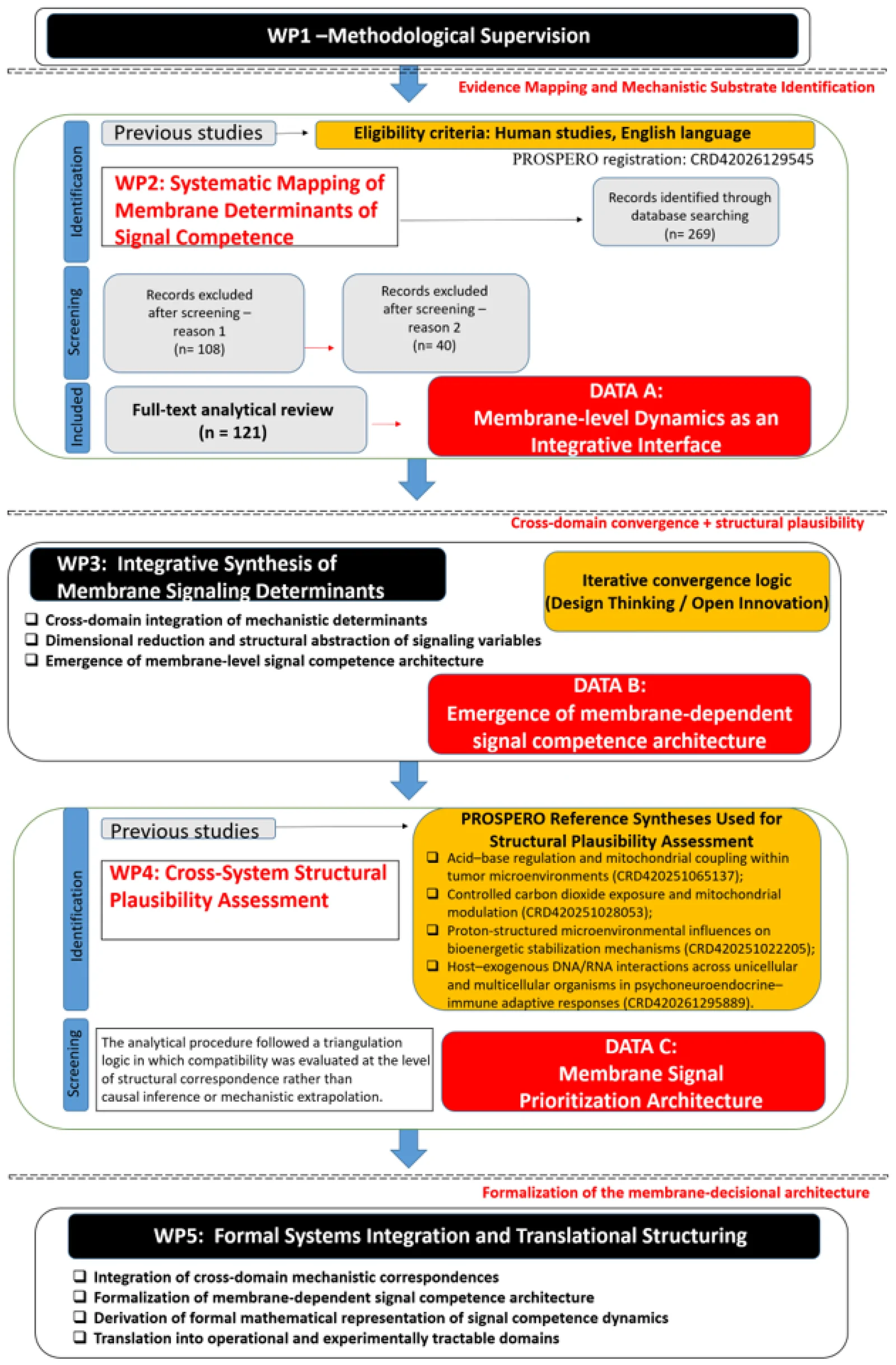
Analytical architecture of the structured synthesis.

This modular analytical structure was designed to preserve inferential discipline, minimize interpretative circularity, and ensure that conceptual conclusions emerged from the integration of mechanistic evidence rather than prior theoretical assumptions. The methodological workflow is summarized in **Figure 1**.

The study design follows a Work Breakdown Structure framework that organizes the research process into sequential analytical stages (Project Management Institute, 2019; Gaspary et al., 2026b). Evidence retrieval and mechanistic mapping are conducted independently of integrative synthesis and conceptual consolidation, allowing the membrane**-**decisional architecture proposed in this work to emerge through progressive cross**-**domain convergence.

### 2.1 WP1: methodological supervision

WP1 functioned as the supervisory layer of the research architecture. Its primary role was to maintain conceptual coherence and methodological consistency across all analytical stages of the study.

At this stage, the scope and analytical boundaries of the synthesis were defined, and the sequential structure of the work packages was established. Evidence acquisition, mechanistic decomposition, and integrative synthesis were maintained as distinct analytical steps to prevent premature conceptual convergence (Hulley et al., 2007).

A progressive search**-**refinement strategy was applied when necessary to focus the evidence base on mechanisms directly relevant to membrane**-**dependent signaling competence. Reporting transparency followed PRISMA recommendations (Page et al., 2021).

Interdisciplinary consultation was incorporated to support interpretative robustness, allowing external domain expertise to challenge preliminary interpretations without influencing the independence of the analytical process (Chesbrough, 2003).

WP1 did not introduce independent datasets or empirical results but functioned as a procedural layer guiding the progression of the synthesis and ensuring that conceptual conclusions remained traceable to experimentally demonstrated mechanisms.

### 2.2 WP2: systematic mapping of membrane determinants of signal competence

WP2 constituted the primary evidence acquisition stage of the analytical architecture. The objective of this stage was to systematically identify experimentally demonstrated mechanisms through which plasma membrane organization influences signaling competence.

The synthesis focused on membrane-dependent determinants of signaling competence, including receptor clustering, membrane organization, electrochemical state, intracellular routing, and compartmental signal conversion (Lingwood and Simons, 2010; Watanabe et al., 2011; Fork et al., 2014; Changede and Sheetz, 2017; Chen et al., 2023; Armstrong et al., 2025). To ensure transparency, WP2 was conducted as a prospectively registered systematic review in International Prospective Register of Systematic Reviews (PROSPERO) (Centre for Reviews and Dissemination, 2026) (CRD42026129545), focusing on membrane-dependent signaling mechanisms with physiological relevance to human cellular systems.

The objective of this stage was to evaluate whether convergent mechanistic evidence supports the interpretation of the plasma membrane as an organizational interface governing signaling competence rather than a passive structural boundary.

#### 2.2.1 Search strategy

A systematic literature search was conducted in PubMed/MEDLINE from database inception to the most recent available date.

A multiaxis search architecture was implemented to capture experimentally demonstrated determinants of membrane-dependent signaling competence across complementary mechanistic domains. Descriptor combinations and study retention are detailed in **Table 1**.

**Table 1:** Structured search architecture and study retention across membrane signaling determinants.

Analytical axis	Descriptor combination	Records identified
Electrochemical state**-**responsiveness	(“membrane potential”) AND (adaptation OR homeostasis OR regulation) AND (hysteresis OR “nonlinear response” OR bistability OR plasticity)	185
Microdomain–receptor recruitment	(“TLR4” OR “TLR3” OR “TLR7” OR “TLR8” OR “TLR9”) AND (“lipid raft” OR caveolae OR “membrane microdomain”) AND (“RNA” OR “DNA” OR “nucleic acid”)	58
Nucleocapture–routing interface	Refined subset emphasizing uptake, adaptor translocation, and compartmental delivery	17
Membrane–cytoskeleton coupling (targeted retrieval)	(“cytoskeleton” AND “membrane signaling”)	9
Total		269

#### 2.2.2 Selection logic

**Figure 2:**
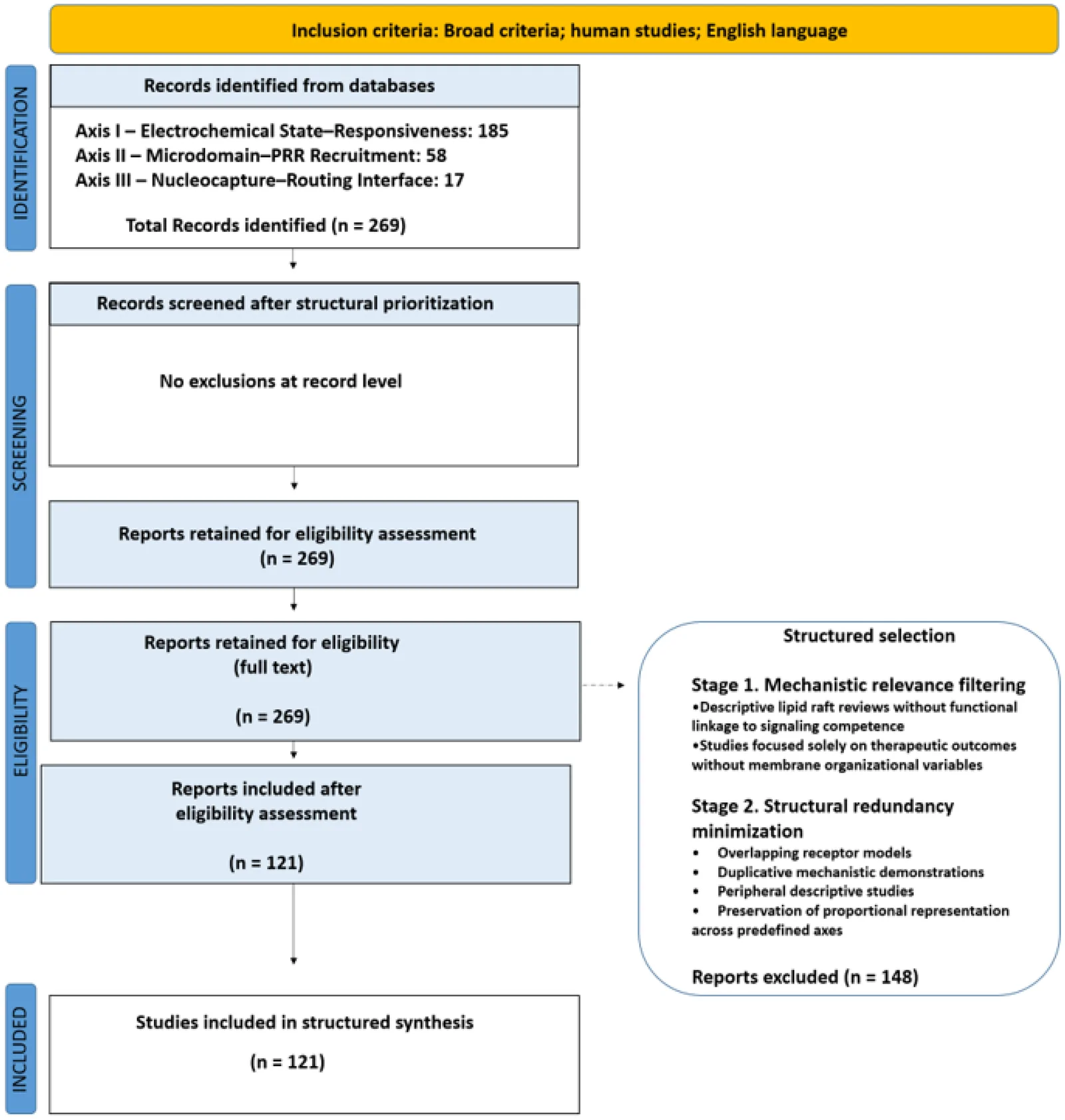
Study selection process with structural prioritization.

Evidence selection followed a two**-**stage filtering procedure prioritizing mechanistic relevance over bibliographic exhaustiveness (Hulley et al., 2007). The overall screening process, including study identification, screening, eligibility assessment, and final inclusion, is summarized in the PRISMA flow diagram (**Figure 2**).

In the initial screening stage, titles and abstracts were evaluated to identify studies reporting experimentally demonstrated membrane-level mechanisms relevant to signaling competence. Studies were retained when they included mechanistic characterization of receptor clustering, membrane microdomain organization, adaptor recruitment, intracellular routing, or compartmental signal conversion. Studies focused exclusively on clinical outcomes without mechanistic resolution or lacking direct membrane-level analysis were excluded.

Full-text assessment was subsequently performed to confirm eligibility based on predefined inclusion criteria, including experimental demonstration of membrane-dependent signaling mechanisms and physiological relevance to human cellular systems.

To minimize overrepresentation of redundant mechanistic models, a second-stage selection prioritized conceptual diversity across studies rather than proportional sampling. Axis I captured broad electrochemical determinants of cellular responsiveness and therefore required wider representation, whereas axes II and III focused on microdomain-dependent receptor organization and routing mechanisms, where a smaller number of studies sufficiently captured mechanistic variation. Targeted inclusion of membrane–cytoskeleton coupling studies ensured representation of structural scaffolding mechanisms relevant to signaling competence.

The final dataset of 121 studies reflects saturation of mechanistic diversity across electrochemical, microdomain**-**dependent, routing**-**dependent, and cytoskeleton**-**associated determinants of membrane signaling competence.

#### 2.2.3 Analytical preparation for integrative synthesis

The retained studies were prepared for integrative synthesis through a structured analytical workflow designed to identify recurrent membrane**-**level determinants of signaling competence.

Initially, experimentally demonstrated mechanisms were decomposed into operational descriptors representing membrane**-**dependent regulatory processes, including receptor clustering, adaptor recruitment, intracellular routing, membrane**-**cytoskeleton coupling, and compartment**-**specific signal conversion.

These descriptors were subsequently standardized into a shared analytical vocabulary to enable cross**-**domain comparison across mechanistically distinct experimental systems.

To maintain analytical consistency and reduce interpretative bias, this preparatory stage followed the structured selection boundaries and reporting criteria defined in the prospectively registered PROSPERO protocol. This ensured that descriptor generation and cross**-**domain comparison remained grounded in experimentally demonstrated mechanisms rather than emerging from *post hoc* interpretative expansion.

This process allowed the evidence base to be reorganized around functional determinants of signaling competence, enabling systematic comparison across heterogeneous biological contexts prior to the integrative synthesis conducted in the subsequent stage.

### 2.3 WP3: integrative synthesis of membrane signaling determinants

The mechanistic corpus identified in WP2 was subjected to structured integrative synthesis to assess whether recurrent membrane**-**level mechanisms converge toward a coherent physiological architecture of signaling competence. This stage represents the transition from evidence mapping to conceptual integration, in which experimentally demonstrated mechanisms are reorganized into higher**-**order functional dimensions to enable systematic cross**-**domain comparison. The synthesis therefore did not organize evidence by receptor families or disease categories but by shared regulatory properties of membrane organization.

Mechanistic variables were reorganized into three complementary dimensions of membrane signaling competence: (i) structural conditions enabling receptor clustering within membrane microdomains; (ii) intracellular routing processes through which clustered receptor complexes are delivered to signal**-**permissive compartments; and (iii) membrane**-**associated constraints—including electrochemical state and cytoskeletal anchoring—that modulate signaling thresholds, spatial confinement, and persistence. These dimensions can be interpreted as emerging from the integrated nanoscale organization of the plasma membrane interface rather than from isolated molecular mechanisms.

This procedure enabled cross**-**domain comparison of heterogeneous experimental findings within a common analytical perspective of membrane**-**dependent signal prioritization, supporting convergence toward a coherent physiological architecture examined in the Results section.

### 2.4 WP4: cross-system structural plausibility assessment

WP4 evaluated whether the membrane**-**level mechanisms identified in WP3 remain physiologically coherent when considered within broader microenvironment**-**dependent stabilization processes.

This stage functioned as a bounded plausibility assessment, examining compatibility between the synthesized membrane architecture and established physiological variables known to influence cellular responsiveness, including acid**-**base dynamics (Mitchell, 1961), redox balance (Covarrubias et al., 2021), membrane organization (Lingwood and Simons, 2010), and mitochondrial coupling (Wallace, 2012).

To support this assessment, structured reference evidence from prospectively registered systematic syntheses in PROSPERO (Page et al., 2021) was used as calibration anchors. These included studies on acid**-**base regulation and mitochondrial coupling in tumor microenvironments (CRD420251065137), controlled carbon dioxide exposure (CRD420251028053), proton**-**structured microenvironmental influences on bioenergetic stabilization (CRD420251022205), and host–exogenous DNA/RNA interactions across biological systems (CRD420261295889).

These registrations were used to guide cross**-**domain comparison rather than to extend the evidence base established in WP2–WP3. They were selected because they represent recent systematic syntheses addressing extracellular microenvironment dynamics, including proton fluctuations, metabolic reprogramming (e.g., Warburg**-**related processes), and multigenomic inputs interacting with cellular systems. As such, they provide relevant reference contexts for evaluating whether the proposed membrane**-**centered framework remains structurally coherent under diverse microenvironmental conditions.

Compatibility was evaluated at the level of structural correspondence rather than causal extrapolation (Belton and Stewart, 2002). No additional mechanisms were introduced, and all interpretations remained constrained by experimentally demonstrated processes identified in earlier stages.

Analytical consistency was supported through divergence–convergence reasoning (Chesbrough, 2003) and structured interdisciplinary consultation (Hulley et al., 2007), ensuring that emerging interpretations remained grounded in mechanistic evidence.

Finally, a heuristic boundary calibration step was applied to define the limits of the proposed framework (Simon, 1977; Gigerenzer and Gaissmaier, 2011). This ensured separation between mechanistic evidence and conceptual synthesis, maintaining the framework as a physiologically plausible, hypothesis**-**derived construct rather than a validated explanatory model.

Importantly, the analytical design explicitly addressed the potential risk of interpretative circularity inherent to hypothesis**-**derived frameworks. As the proposed architecture aims to organize an already observed but underintegrated phenomenon, the evaluation was constrained to structural coherence across independently established mechanistic domains rather than attempting direct validation of the conceptual construct itself.

This approach ensured that the framework was not assessed based on its own internal logic but on its consistency with experimentally demonstrated processes derived from heterogeneous biological contexts. In this sense, the plausibility assessment operated as a safeguard against circular reasoning, preserving inferential discipline while allowing conceptual integration.

### 2.5 Formal system integration and translational structuring

WP5 constituted the final integration stage of the analytical architecture, consolidating the evidence base from WP2, the integrative synthesis from WP3, and the structural calibration from WP4 into a system**-**level interpretation of membrane**-**dependent signaling competence.

No additional empirical datasets were introduced at this stage. Instead, previously established mechanistic constructs were integrated into a coherent multiscale framework within the inferential boundaries defined in earlier stages. Recurrent relationships across membrane organization, receptor clustering, routing processes, and compartmental signal conversion were abstracted into a structured representation of signaling competence.

This abstraction enabled a formal representation of the relationship between signal availability, membrane competence, and intracellular routing dynamics. This formulation is not intended as a predictive model but as a structured representation of the architecture emerging from the integrative synthesis.

The structural domains identified in this stage emerged through progressive abstraction of recurrent stabilization variables observed across membrane dynamics, routing mechanisms, electrochemical states, cytoskeletal constraints, and bioenergetic conditions (Mitchell, 1961; Wallace, 2012).

To ensure translational coherence, the framework was examined using established research design criteria, including Specific, Measurable, Achievable, Relevant, and Time-bound (SMART) (Doran, 1981) and Feasible, Interesting, Novel, Ethical, and Relevant (FINER) (Project Management Institute, 2021) principles. This evaluation served as a structured assessment of conceptual robustness and methodological coherence rather than empirical validation.

#### 2.5.1 Derivation of operational implications from membrane-decisional architecture

The integrative framework developed in WP5 provides a structured basis for articulating logically consistent interpretations of membrane**-**dependent signaling behavior.

Within this context, the operational implications presented in this study reflect coherent expressions of the relationships identified across membrane organization, clustering dynamics, routing processes, and physicochemical constraints. These interpretations are formulated to preserve internal consistency with the synthesized evidence while extending the conceptual architecture toward experimentally addressable conditions.

Accordingly, the statements derived from this framework are not intended as validated predictions but as logically grounded propositions emerging from integrative synthesis. Their role is to make explicit the implications of the proposed architecture, allowing them to be examined, challenged, and refined in future experimental work.

## 3 Results

The structured synthesis conducted across WP2–WP5 identified a recurrent organizational pattern linking membrane microdomain architecture (Lingwood and Simons, 2010), receptor clustering dynamics (Changede and Sheetz, 2017), intracellular routing mechanisms (Watanabe et al., 2011; Fork et al., 2014), and cytoskeleton**-**associated constraints (Heikkinen et al., 2009) to the emergence of signaling competence.

Across heterogeneous experimental systems, these mechanisms converged toward a shared regulatory principle: extracellular signals acquire functional intracellular relevance only when membrane organizational conditions permit clustering, routing, and compartmental conversion within signal**-**permissive domains. This supports the interpretation of the plasma membrane as a signal prioritization interface governing the transition from signal presence to signaling competence.

Results are presented following the analytical structure defined in Section 2. WP2 identifies the primary membrane**-**level determinants of signaling competence, WP3 examines their integrative convergence, WP4 evaluates cross**-**system structural plausibility, and WP5 consolidates these findings into a system**-**level interpretation.

### 3.1 Results of WP2: structural determinants of membrane-level signal competence

WP2 generated the primary mechanistic map of membrane**-**level determinants associated with signaling competence. Across the retained corpus, extracellular signal presence and functional signaling output emerged as distinct biological conditions, with this discrepancy consistently associated with structural properties of plasma membrane organization.

The results of WP2 are presented in two complementary layers. First, the mapped literature identifies the plasma membrane as a state**-**dependent interface governing whether extracellular inputs become biologically effective. Second, the retained studies indicate that microdomain organization and receptor clustering represent the principal structural determinants through which this interface acquires regulatory specificity.

#### 3.1.1 The plasma membrane as a signaling competence interface

The systematic mapping conducted in WP2 revealed a consistent pattern across heterogeneous experimental domains: extracellular ligand presence does not reliably translate into functional cellular responses. Across retained studies spanning innate immune sensing, viral nucleic**-**acid detection, metabolic receptor signaling, neuronal excitability, and epithelial regulation, ligand binding at the cell surface was repeatedly shown to be insufficient to guarantee downstream transcriptional activation or measurable physiological output.

Experimental observations demonstrate that membrane potential states and ion**-**channel**-**dependent modulation of cellular excitability strongly influence whether extracellular inputs trigger intracellular signaling cascades (Amarillo et al., 2008; Cavaliere et al., 2012; Podda and Grassi, 2014; Kuo et al., 2020). Complementary evidence indicates that receptor engagement can remain functionally silent unless membrane**-**associated trafficking and compartmental routing enable signal propagation toward intracellular signaling domains (Watanabe et al., 2011; Gabriel et al., 2012; Zuniga et al., 2024). Together, these findings establish that extracellular signal presence and signaling competence represent distinct biological conditions.

A central observation across the corpus was that variation in signaling outcome frequently occurred in the absence of major changes in receptor abundance or ligand availability. Instead, decisive differences were associated with membrane**-**level organizational states. Ion channels and membrane**-**associated transport systems dynamically modulate membrane potential and intracellular signaling thresholds, thereby shaping cellular responsiveness (Zuniga et al., 2024; Bhoi et al., 2025). In parallel, mitochondrial membrane potential acts as a bioenergetic regulator influencing calcium signaling, reactive oxygen species production, and compartmentalized responses (Ahmed Selim and Wojtovich, 2025). In neuronal and excitable systems, membrane depolarization and hyperpolarization states alter receptor responsiveness and downstream transcriptional outputs without requiring changes in receptor expression levels (Sun et al., 2020; Dudai et al., 2022; Tiwari et al., 2023).

The retained corpus further indicates that plasma membrane organization operates as a state**-**dependent regulatory substrate rather than a passive structural boundary. Membrane microdomains and lipid raft structures emerge as localized control environments in which receptors, adaptor proteins, kinases, cytoskeletal elements, and trafficking machinery assemble into dynamic signaling platforms (Li et al., 2004; Smythies et al., 2010; Abate et al., 2020). In immune and epithelial systems, cholesterol**-**dependent microdomain organization regulates Toll**-**like receptor recruitment, activation thresholds, and downstream inflammatory signaling (Sun et al., 2009; Koarai et al., 2012; Liu et al., 2016). Similarly, cytoskeleton**-**associated scaffolding stabilizes membrane signaling complexes and facilitates receptor clustering required for effective signal propagation (Yin and Stull, 1999; Heikkinen et al., 2009).

#### 3.1.2 Microdomain organization and receptor clustering as primary structural determinants

A second major result of WP2 was the recurrent identification of membrane microdomain organization as a primary structural determinant of signaling competence. Across the retained mechanistic literature, the plasma membrane did not behave as a homogeneous signaling surface in which receptor activation depends solely on ligand availability or receptor density. Instead, convergent experimental evidence showed that many signaling systems operate within cholesterol**-** and sphingolipid**-**enriched nanodomains that create spatially restricted environments for receptor assembly and signal propagation (Boggs et al., 2004; Li et al., 2004; Liu et al., 2016).

Within these domains, receptor clustering consistently emerged as a prerequisite for productive signaling activation. Studies examining adhesion receptor assemblies demonstrated that nanoscale clusters of integrins and cadherins function as modular signaling units capable of sustaining cooperative signaling events within confined membrane regions (Changede and Sheetz, 2017). Similar clustering**-**dependent activation mechanisms were observed in oncogenic signaling systems, where the formation of membrane**-**associated receptor supercomplexes triggered downstream kinase recruitment and cytoskeletal remodeling required for malignant signaling amplification (Guerra et al., 2022). Together, these findings indicate that receptor engagement becomes biologically consequential only when supported by structurally competent membrane organization.

The corpus further demonstrated that perturbation of microdomain integrity frequently altered signaling output without necessarily modifying ligand binding itself. Cholesterol enrichment or depletion of the plasma membrane has been shown to directly influence Toll**-**like receptor activation by modulating receptor recruitment into lipid raft domains (Sun et al., 2009; Huang et al., 2015). Similarly, disruption of lipid raft architecture through cholesterol efflux mechanisms altered macrophage inflammatory signaling by reorganizing membrane receptor distribution (Smythies et al., 2010). These observations indicate that lipid composition and nanoscale membrane organization establish the structural conditions required for receptor**-**mediated signal amplification.

In several experimental systems, membrane microdomains also functioned as regulatory gateways controlling ligand delivery to intracellular signaling compartments. Lipid raft**-**associated proteins such as Raftlin were shown to mediate cellular uptake and endosomal delivery of nucleic-acid ligands required for Toll**-**like receptor activation (Watanabe et al., 2011). Similarly, flotillin**-**dependent membrane domains were found to facilitate the internalization and trafficking of nucleic**-**acid ligands to intracellular receptors, thereby enabling productive innate immune signaling (Fork et al., 2014). These findings demonstrate that membrane organization influences signaling competence not only through receptor clustering but also through routing of extracellular ligands toward signal**-**permissive intracellular compartments.

Additional evidence within the WP2 corpus revealed that membrane**-**associated extracellular matrix and lipid scaffolds can further regulate receptor accessibility and ligand routing. For example, extracellular matrix proteins such as lumican were shown to interact with lipid raft**-**associated receptors and modulate the balance between Toll-like receptor 4 (TLR4) and Toll-like receptor 9 (TLR9) signaling through differential ligand trafficking (Maiti et al., 2021). Likewise, cholesterol metabolism and lipid remodeling processes have been shown to influence immune signaling pathways by regulating the membrane distribution and activation thresholds of receptor complexes (Bibby et al., 2020).

Importantly, several studies indicated that membrane microdomains act as dynamic assembly zones in which receptors, adaptor complexes, and cytoskeletal stabilizing elements transiently converge to generate signaling competence. Proteomic analyses of lipid raft fractions demonstrated the coexistence of receptors, trafficking proteins, and cytoskeleton**-**associated elements within the same nanoscale membrane environments (Li et al., 2004). These domains therefore function as transient organizational hubs in which receptor proximity, scaffold recruitment, and signaling persistence can be locally regulated.

Across the WP2 corpus, the repeated observation that receptor activation depends on nanoscale membrane organization rather than receptor availability alone suggests the existence of a structural regulatory layer upstream of classical receptor–ligand models. Receptors may remain ligand**-**bound yet functionally weak when clustering thresholds are not achieved, whereas receptors assembled within competent membrane microdomains can generate amplified signaling responses even under modest ligand exposure.

Taken together, the mechanistic evidence identified in WP2 supports the interpretation that membrane microdomain organization and receptor clustering constitute the principal structural determinants through which the plasma membrane regulates signaling competence. Rather than acting solely as a passive surface hosting receptors, the plasma membrane operates as a spatially structured regulatory interface that determines whether extracellular signals remain surface**-**bound events or transition into biologically effective intracellular signaling responses.

This structural layer provided the mechanistic basis for the integrative synthesis conducted in WP3, which examined how clustering**-**dependent competence is extended through membrane**-**governed intracellular routing and compartmental signal conversion.

### 3.2 Results of WP3: routing competence and endocytic signal conversion

While membrane microdomains provide the structural conditions necessary for receptor clustering, the integrative synthesis conducted in WP3 demonstrated that clustering alone is not sufficient to generate productive signaling responses. Across the mechanistic corpus analyzed in WP2, a second regulatory layer consistently emerged through membrane**-**dependent routing mechanisms that determine whether clustered receptor–ligand complexes are delivered to intracellular compartments capable of supporting downstream signaling conversion.

In numerous signaling systems, productive downstream responses required controlled trafficking of receptor complexes through endocytic pathways. Following ligand engagement and receptor clustering at the plasma membrane, signaling complexes frequently underwent internalization through clathrin**-**mediated endocytosis, caveolar pathways, or related membrane trafficking processes. Experimental studies demonstrated that nucleic**-**acid ligands can be actively internalized through membrane**-**associated uptake complexes, enabling delivery to intracellular signaling compartments (Vidakovics et al., 2010; Singh et al., 2013). Similarly, receptor–ligand complexes internalized through macropinocytosis or clathrin**-**dependent mechanisms were shown to activate intracellular nucleic**-**acid sensing pathways following delivery to endosomal compartments (Kleinman et al., 2012; Inoue et al., 2020). These findings indicate that receptor engagement at the plasma membrane frequently represents only the initial stage of signaling activation.

Consistent with this interpretation, disruption of trafficking pathways prevented downstream signaling activation even when receptor binding and surface clustering remained intact. In immune signaling systems, intracellular routing of nucleic-acid ligands to endosomal Toll-like receptors was required for adaptor recruitment and transcriptional activation (Perego et al., 2018; Obermann et al., 2022). Similarly, internalization of extracellular vesicles containing nucleic acids or microbial components enabled ligand delivery to intracellular receptors that initiate inflammatory signaling cascades (Ebihara et al., 2008; Vidakovics et al., 2010). These observations demonstrate that membrane-governed routing competence determines whether extracellular signals reach intracellular signaling platforms capable of sustaining transcriptional activation.

Conversely, alternative routing trajectories frequently attenuated signaling responses. Lysosomal degradation, receptor recycling, ubiquitin**-**mediated turnover, or incomplete compartmental delivery redirected engaged receptor complexes toward functionally silent or transient outcomes. Experimental studies demonstrated that regulated endocytosis of membrane channels or receptors dynamically alters signaling amplitude by modifying the number of surface**-**accessible signaling complexes (Gabriel et al., 2012; Han et al., 2018). Similarly, alterations in lipid metabolism and membrane cholesterol distribution modulate immune signaling pathways by influencing receptor localization and intracellular trafficking dynamics (Sun et al., 2009; Georgiadi et al., 2013; Bibby et al., 2020).

Importantly, receptor–ligand interactions that do not satisfy membrane**-**dependent competence conditions are not biologically inert. Such interactions may follow alternative trajectories, including receptor recycling, degradation through lysosomal pathways, or subthreshold signaling that does not propagate into transcriptional activation. These outcomes reflect the conditional nature of signaling competence, in which membrane organization determines not only signal amplification but also attenuation and selective filtering of extracellular inputs.

Additional evidence indicates that routing competence is further modulated by membrane electrical and structural states. Ion channels regulating membrane potential shape cellular responsiveness by controlling calcium influx, synaptic transmission, and downstream signaling activation (Amarillo et al., 2008; Cavaliere et al., 2012; Jeremic et al., 2021). In neuronal systems, plastic regulation of potassium and calcium channels dynamically alters membrane excitability and influences the conversion of receptor activation into intracellular signaling responses (Ng et al., 2008; Podda and Grassi, 2014; Bhoi et al., 2025).

Membrane architecture also constrains the spatial propagation of signaling events. Experimental measurements of membrane tension dynamics demonstrated that perturbations remain confined to localized regions rather than propagating across the entire plasma membrane surface (Shi et al., 2018). Cytoskeleton**-**associated transmembrane structures limit diffusion of mechanical perturbations, resulting in spatially confined activation of mechanosensitive signaling pathways. This spatial confinement indicates that signaling responses emerge within localized membrane domains rather than through uniform propagation across the cell surface.

Complementary evidence further illustrates how spatial routing contributes to signaling competence. Biochemical reconstitution experiments demonstrated that signaling regulators such as Rho family small GTPases undergo tightly controlled transitions between cytosolic and membrane**-**associated states, with membrane insertion preceding activation and subsequent stabilization through effector interactions (Armstrong et al., 2025). These spatially patterned transitions generate localized signaling domains that regulate cytoskeletal dynamics during processes such as cell migration, morphogenesis, and wound repair. Consistent with this architecture, membrane–cytoskeleton coupling stabilizes receptor organization and constrains signal propagation by regulating receptor mobility and clustering dynamics (Yin and Stull, 1999; Heikkinen et al., 2009), while nanoscale adhesion receptor clusters sustain cooperative signaling within confined membrane domains (Changede and Sheetz, 2017). Computational models further support that membrane potential**-**dependent structural plasticity can dynamically reorganize signaling connectivity within cellular systems (Crook et al., 2007; Solís-Perales and Estrada, 2021).

Taken together, the WP3 synthesis indicates that membrane**-**dependent routing mechanisms constitute a second integrative layer of signaling regulation. Productive signaling emerges only when receptor engagement, microdomain clustering, and intracellular routing converge within signal**-**permissive domains. Under this interpretation, the plasma membrane regulates not only the spatial assembly of receptor complexes but also the trafficking trajectories and structural constraints that determine whether extracellular signals acquire functional regulatory significance within the cell.

Collectively, these observations indicate that membrane**-**dependent signal prioritization emerges from a spatially organized, nanoscale biological interface integrating structural, electrochemical, and trafficking determinants. Within this interpretation, the plasma membrane can be understood as a functional nanobiome, in which signaling competence is not a property of isolated molecular interactions but of the dynamically organized membrane environment in which they are embedded.

### 3.3 Results of WP4: multigenomic signal prioritization at the membrane interface

The analysis conducted in WP4 examined whether the membrane**-**level mechanisms identified in WP2 and WP3 remain structurally coherent when situated within broader physiological conditions known to modulate cellular responsiveness. Rather than expanding the evidentiary base, this stage focused on evaluating whether the proposed membrane**-**centered architecture maintains internal consistency when confronted with independently established biological systems.

Across the reference contexts considered, a consistent pattern was observed: the structural determinants of signaling competence—namely membrane microdomain organization and routing**-**dependent signal delivery—remained compatible with core physiological variables governing cellular behavior. These included acid**-**base dynamics (Chesbrough, 2003), redox regulation (Covarrubias et al., 2021), membrane organizational states (Lingwood and Simons, 2010), and mitochondrial coupling processes (Wallace, 2012).

The planned cross**-**domain confrontations with prospectively registered systematic syntheses (PROSPERO) (Centre for Reviews and Dissemination, 2026) are presented in detail in the subsequent section.

### 3.4 Results of WP5: emergence of membrane-decisional architecture

The cross**-**domain synthesis conducted across WP2–WP4 revealed a consistent organizational pattern linking membrane structure, receptor clustering dynamics, intracellular routing mechanisms, and compartmental signal conversion to the emergence of signaling competence. Taken together, these findings support the interpretation that plasma membrane organization implements a regulatory layer operating upstream of classical intracellular signal transduction pathways (Li et al., 2004; Changede and Sheetz, 2017; Shi et al., 2018).

Within this layer, extracellular signals become biologically effective only when a defined sequence of membrane**-**governed conditions is satisfied. These include the following: (i) access to competent membrane domains, (ii) receptor clustering within structurally permissive microdomains, (iii) routing competence enabling intracellular trafficking toward signaling compartments, and (iv) successful compartmental conversion into transcriptionally active signaling complexes. Experimental evidence demonstrates that ligand engagement alone is insufficient unless these conditions are met, particularly in systems requiring endosomal activation and adaptor recruitment (Watanabe et al., 2011; Kleinman et al., 2012; Obermann et al., 2022).

These processes collectively define membrane-decisional architecture as the integrated set of membrane-dependent mechanisms governing which extracellular signals acquire signaling competence within cellular regulatory networks. Rather than functioning as a passive boundary, the plasma membrane operates as a dynamic prioritization interface, regulating signal selection through receptor clustering (Sun et al., 2009; Smythies et al., 2010; Huang et al., 2015), routing competence (Han et al., 2018; Armstrong et al., 2025), and compartmental signal conversion (Han et al., 2018; Obermann et al., 2022). Under conditions in which multiple extracellular inputs converge at the same membrane interface, these integrated processes determine which signals acquire regulatory competence and which remain biologically silent despite surface engagement (Georgiadi et al., 2013; Bibby et al., 2020). This interpretation does not imply agency or autonomous control by exogenous elements; rather, membrane-decisional architecture represents a host-implemented interface through which heterogeneous extracellular inputs are selectively integrated into cellular regulatory networks.

#### 3.4.1 Operational derivation from membrane-decisional architecture

Operational domains were defined at the level of system organization, reflecting recurrent determinants identified across WP2–WP4. Only constructs demonstrating cross**-**stage convergence, compatibility with experimentally established membrane**-**level mechanisms, and consistency with physicochemical constraints were retained.

These domains represent coordinated expressions of membrane**-**dependent signal prioritization, in which clustering, routing, and state**-**dependent constraints collectively regulate the transition from extracellular signal presence to signaling competence.

The operational domains summarized in **Table 2** are grounded in experimentally demonstrated mechanisms extensively described in membrane biology and cellular signaling literature, including mechanisms related to membrane microdomain organization and lipid raft dynamics (Li et al., 2004; Lingwood and Simons, 2010), receptor clustering and mechanotransduction processes (Changede and Sheetz, 2017), membrane**-**dependent routing and trafficking dynamics (Watanabe et al., 2011; Gabriel et al., 2012; Han et al., 2018), and bioenergetic modulation of signaling through mitochondrial coupling and redox regulation (Rich and Maréchal, 2010; Verdin, 2015; Centre for Reviews and Dissemination, 2026). These domains therefore represent structured abstractions of recurrent mechanistic patterns rather than independently proposed variables.

**Table 2:** Operational domains and corresponding experimental readouts.

Operational domain	Structural determinant	Membrane-level mechanism	Measurable readout
Signal routing competence	Membrane microdomain organization	Adaptor recruitment and endocytic routing dynamics	Transcriptional output (e.g., cytokine signaling, IFN response)
Microdomain clustering thresholds	Cholesterol-dependent membrane organization	Receptor clustering probability and nanoscale domain formation	Signal amplification, receptor activation assays
Threshold-dependent responsiveness	Membrane electrochemical state	Ion-channel**-**mediated modulation of activation thresholds	Responsiveness to perturbation, activation sensitivity curves
Signal amplitude and persistence	Lipid composition and membrane remodeling	Regulation of receptor localization and trafficking stability	Duration and intensity of signaling output
State-dependent signal prioritization	Integrated membrane organizational state	Combined clustering and routing constraints under heterogeneous inputs	Differential pathway activation under identical ligand exposure
Bioenergetic modulation of signaling	Mitochondrial coupling and redox balance	Regulation of intracellular signaling thresholds and routing efficiency	ROS levels, NAD^+^/NADH ratio, metabolic signaling markers
Spatial confinement of signaling	Membrane–cytoskeleton coupling	Restriction of signal propagation and localized activation domains	Spatial signaling gradients, localized activation assays
Multigenomic signal integration	Convergent host–exogenous signaling inputs	Membrane-mediated filtering of heterogeneous molecular signals	Differential transcriptional response to mixed-signal environments

**Figure 3:**
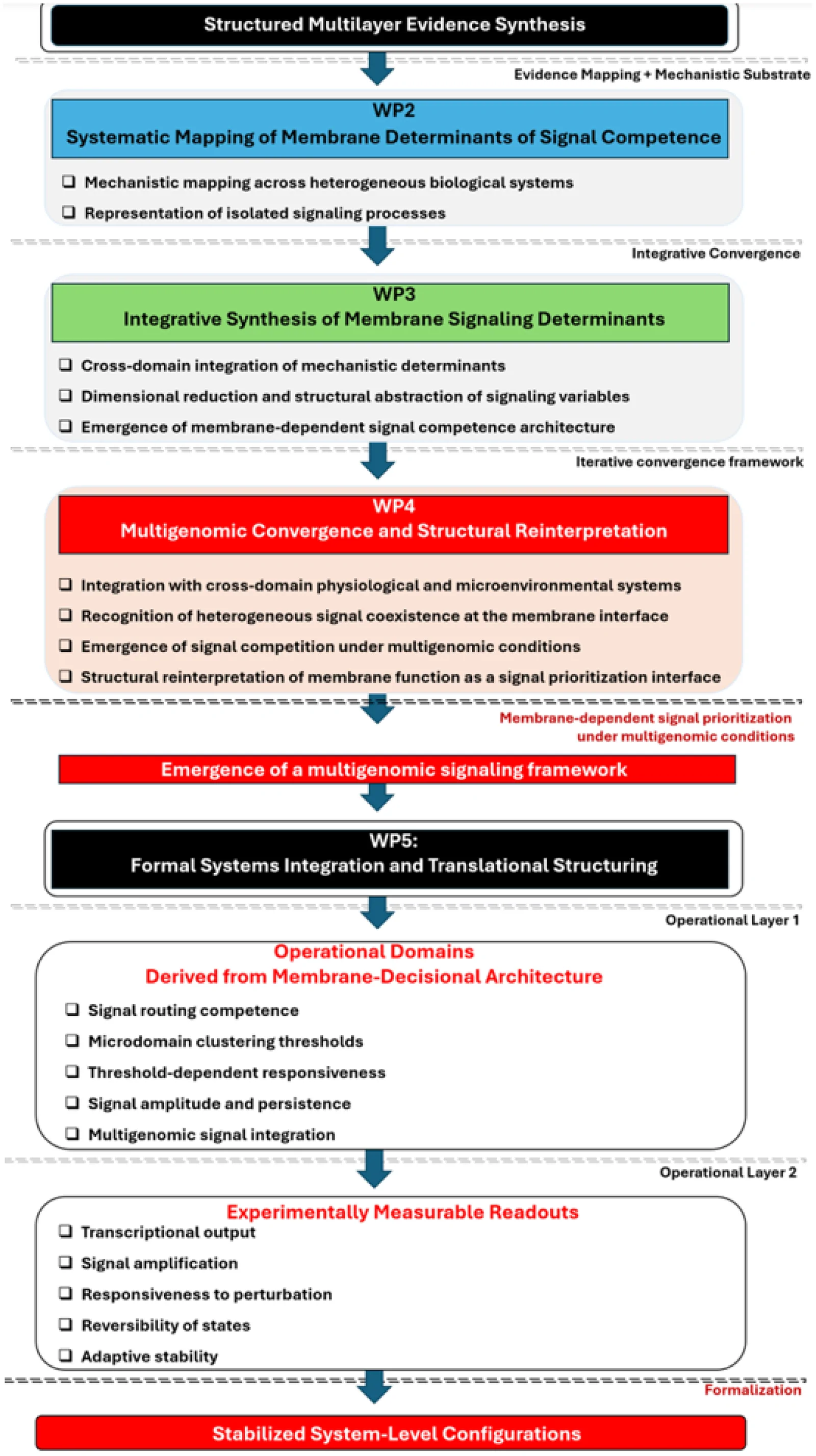
From multigenomic signal coexistence to membrane-dependent signal prioritization: an operationalization pipeline.

These operational implications are not intended as predictive models in isolation, but as structured expressions of the architecture derived from the integrative synthesis (**Figure 3**). Their function is to bridge conceptual organization and empirical investigation by enabling experimental exploration of signaling competence thresholds, routing dynamics, and state**-**dependent responsiveness.

This schematic illustrates the progression from structured evidence synthesis to experimentally tractable system**-**level outcomes. WP2 maps membrane**-**level determinants of signaling competence across heterogeneous biological systems, while WP3 integrates these determinants through cross**-**domain synthesis and structural abstraction, enabling the emergence of a membrane**-**dependent signal competence architecture.

WP4 represents the critical conceptual transition in which integration across physiological and microenvironmental domains reveals the coexistence of heterogeneous signals and the emergence of signal competition under multigenomic conditions. This step reframes membrane organization as a signal prioritization interface rather than a passive signaling platform.

WP5 consolidates this architecture through formal systems integration, including the derivation of a mathematical representation of signal competence and its translation into operational domains. These domains are subsequently linked to experimentally measurable readouts, including transcriptional output, responsiveness to perturbation, reversibility of states, and adaptive stability.

Together, this pipeline demonstrates how membrane**-**dependent mechanisms translate multigenomic signal coexistence into structured prioritization and stabilized physiological configurations.

#### 3.4.2 Applying translational structuring: SMART and FINER alignment

To assess the scientific tractability of the membrane**-**decisional architecture framework, the proposed model was examined using established research design criteria, including SMART (Doran, 1981) and FINER (Hulley et al., 2007) principles. The resulting alignment between the framework and these criteria is summarized in **Table 3**.

**Table 3:** SMART and FINER criteria.

Criterion	Interpretation within the membrane-decisional architecture framework	How the criterion is satisfied
SMART framework		
Specific	The framework focuses on membrane-level determinants of signaling competence, including microdomain organization, receptor clustering thresholds, routing competence, and compartmental signal conversion.	Research questions can be formulated around measurable membrane variables such as microdomain integrity, receptor clustering density, adaptor recruitment, and endocytic routing dynamics.
Measurable	Signaling competence can be operationalized through experimentally observable parameters such as receptor clustering, adaptor translocation, intracellular trafficking, and transcriptional output following ligand engagement.	Established experimental methods (super-resolution microscopy, lipid perturbation assays, trafficking inhibition models, and signaling readouts) allow direct measurement of these variables.
Achievable	The mechanisms proposed are already experimentally observable in membrane biology and cell signaling studies.	Existing *in vitro*, cellular, and biochemical systems allow controlled manipulation of membrane organization, lipid composition, and trafficking pathways.
Relevant	The framework addresses a persistent discrepancy in cell signaling: ligand presence does not always generate signaling competence.	Provides a physiologically coherent explanation for context-dependent signaling responses across immune, metabolic, and receptor signaling systems.
Time-bound	Membrane-dependent signal competence can be evaluated across defined temporal windows of receptor engagement, clustering, internalization, and transcriptional activation.	Time-resolved imaging and signaling assays allow dynamic observation of transitions from ligand engagement to intracellular signaling output.
FINER framework		
Feasible	The framework relies on experimentally established mechanisms rather than novel technological requirements.	Studies can be conducted using existing tools in membrane biophysics, receptor trafficking research, and cell signaling analysis.
Interesting	The model integrates findings from membrane biology, receptor trafficking, immunology, and systems physiology into a unified interpretation of signaling competence.	Offers a conceptual bridge between membrane organization and adaptive cellular responses across physiological systems.
Novel	Introduces the concept of membrane-decisional architecture as an organizing interpretation of membrane-dependent signal prioritization.	Reframes established membrane mechanisms within a coherent physiological architecture explaining signal competence.
Ethical	The framework does not require ethically problematic experimental manipulation.	Investigations can be performed using standard cellular models and existing experimental systems.
Relevant	The concept addresses fundamental questions regarding how cells integrate heterogeneous extracellular signals.	Provides a physiologically grounded model for studying signal prioritization in multigenomic signaling environments.

Taken together, the cross**-**domain synthesis conducted across WP2–WP5 establishes a coherent mechanistic and operational foundation for interpreting plasma membrane organization as a regulatory interface governing signaling competence within human cellular systems. This integrated architecture links structural determinants, routing dynamics, and multigenomic signal environments into a unified framework capable of supporting both conceptual interpretation and experimentally tractable investigation. The broader physiological implications of this membrane**-**dependent signal prioritization architecture are examined in the following section.

## 4 Membrane-dependent signal prioritization within microenvironmental and multigenomic physiological systems

Building upon the mechanistic synthesis presented in the preceding sections, this stage examines the physiological implications of membrane-dependent signal prioritization within complex biological systems. The integrative analysis indicates that plasma membrane organization functions as a dynamic interface through which extracellular molecular inputs either acquire or fail to acquire signaling competence. Through microdomain-dependent receptor clustering, endocytic routing, and compartmental signal conversion, membrane architecture conditions which extracellular signals become capable of influencing intracellular regulatory networks.

Converging evidence demonstrates that membrane organization regulates receptor clustering, spatial availability of signaling complexes, intracellular trafficking, and signal amplification thresholds (Simons and Ikonen, 1997; Brown and London, 2000; Pike, 2006; Lingwood and Simons, 2010; Watanabe et al., 2011; Gabriel et al., 2012; Changede and Sheetz, 2017). Within this framework, extracellular signal presence and functional signaling output represent distinct conditions, with membrane-dependent processes governing the transition between them.

Accordingly, cellular responses reflect not the total set of extracellular signals, but the subset that successfully achieves signaling competence at the membrane interface. In this sense, the plasma membrane operates as a biological filtering system that determines which inputs are permitted to enter intracellular regulatory pathways. This filtering is not passive but emerges from the coordinated interaction between membrane organization, trafficking dynamics, and local physicochemical conditions (Sun et al., 2009; Huang et al., 2015; Han et al., 2018).

This perspective is formalized here as Membrane Decision Theory, in which heterogeneous molecular inputs compete for signaling competence under spatial, energetic, and structural constraints. Rather than responding indiscriminately to all extracellular stimuli, cells respond selectively to signals that satisfy the conditions required for clustering competence, routing competence, and compartmental conversion (Changede and Sheetz, 2017; Shi et al., 2018).

Importantly, this prioritization process unfolds within intrinsically heterogeneous and multigenomic signaling environments. Human physiology operates within a composite molecular landscape in which host-derived mediators coexist with microbiota-associated metabolites, viral nucleic acids, extracellular vesicle cargo, and environmentally modulated inputs. These signals interact with shared membrane interfaces, where receptor recruitment, internalization, and intracellular routing determine their capacity to influence regulatory networks (Qin et al., 2010; Vidakovics et al., 2010; Singh et al., 2013; Handley, 2016; Sender et al., 2016; Pertea et al., 2018; Liang and Bushman, 2021; Obermann et al., 2022).

Within such environments, signaling competence becomes a conditional property rather than a direct consequence of ligand availability. Membrane-dependent internalization, trafficking dynamics, and local organizational states determine whether extracellular inputs are propagated, attenuated, or remain functionally silent, contributing over time to the stabilization of adaptive physiological configurations through membrane excitability, ion channel regulation, and intracellular signaling thresholds (Amarillo et al., 2008; Cavaliere et al., 2012; Jeremic et al., 2021). Consistent with the cross-system structural calibration established in WP4, the following subsections examine how this membrane-dependent architecture remains structurally compatible with key physiological stabilization domains, including acid-base regulation, gaseous microenvironmental modulation, proton-dependent bioenergetics, and multigenomic host–exogenous signaling interactions.

### 4.1 Acid-base regulation and mitochondrial coupling as structural calibration domains

One of the most fundamental physicochemical variables influencing cellular signaling behavior is the regulation of proton gradients and acid**-**base balance across biological membranes. Variations in extracellular and intracellular pH directly affect membrane electrochemical states, receptor conformational dynamics, ion channel activity, and mitochondrial coupling efficiency. For this reason, acid-base regulation provides a critical physiological domain for evaluating whether the membrane-dependent signal prioritization architecture remains coherent under conditions that modulate bioenergetic and electrochemical states.

Within cellular systems, proton gradients play a central role in maintaining mitochondrial oxidative phosphorylation through the chemiosmotic coupling mechanism originally described by Mitchell (1961). Proton flux across the inner mitochondrial membrane generates the electrochemical potential required to drive Adenosine triphosphate (ATP) synthesis, linking cellular energy production to membrane electrochemical states. Subsequent analyses further demonstrate how proton transfer across membrane-bound complexes establishes the energetic conditions required for metabolic homeostasis (Rich and Maréchal, 2010).

Beyond ATP synthesis, mitochondrial coupling efficiency and intracellular proton gradients exert broader regulatory influence over cellular physiology. Mitochondrial metabolism is tightly coupled to redox regulation, Nicotinamide adenine dinucleotide (NAD^+^) availability, and metabolic signaling networks coordinating cellular responses to energetic demand and environmental stress (Verdin, 2015; Yoshino et al., 2018; Covarrubias et al., 2021). These processes are further integrated with redox homeostasis and stress-response pathways, reinforcing the coupling between proton dynamics, bioenergetic state, and cellular signaling behavior (Wang, 2012; Abrescia et al., 2020).

At the level of the plasma membrane, variations in acid-base balance directly influence signaling competence. Changes in extracellular pH alter receptor conformational stability, ion channel conductance, and ligand–receptor interaction dynamics, thereby modifying the probability that extracellular signals achieve effective intracellular activation (Casey et al., 2010; Webb et al., 2011; Sinning and Hübner, 2013). In this context, acid-base regulation does not act as a signaling input per se, but as a modulator of the conditions under which signaling becomes possible.

This observation introduces an important constraint: the biological effect of extracellular signals cannot be understood independently of the membrane-dependent conditions that determine their conversion into intracellular responses. In environments where proton dynamics significantly alter membrane electrochemical states, identical extracellular signals may produce divergent outcomes depending on the underlying competence of the membrane interface.

This relationship implies that the effective cellular response cannot be described solely as a function of signal availability. Instead, it necessarily depends on the interaction between signal magnitude and membrane-dependent competence, such that:


$$R=S*C_{m},$$

Here, the effective response (*R*) emerges from the interaction between extracellular signal availability (*S*) and a membrane competence term (*C*_m_), reflecting the structural and electrochemical conditions required for signaling activation. The multiplicative formulation reflects the conceptual assumption that membrane competence functions as a permissive constraint on signaling, such that effective responses may remain limited even under high signal availability when membrane competence is low.

Within this formulation, acid-base regulation operates as a primary modulator of *C*_m_. Variations in proton gradients and membrane electrochemical states alter receptor stability, clustering probability, and signaling thresholds, thereby shifting the conditions under which extracellular inputs become biologically effective.

Within the structural calibration logic adopted in WP4, this domain therefore provides a critical test of physiological compatibility. If membrane-dependent signal prioritization constitutes a valid organizing principle, it should remain coherent under conditions in which proton dynamics and acid-base regulation significantly modulate membrane competence.

Evidence from inflammatory and metabolically active microenvironments further supports this interpretation. Chronic inflammatory states and metabolically active tissues frequently exhibit altered redox balance, metabolic flux, and extracellular acidification, conditions that reshape signaling landscapes and influence immune and metabolic regulation (Nathan and Ding, 2010; Franceschi et al., 2017; Hotamisligil, 2017).

Within this framework, acid-base regulation can therefore be understood as a fundamental calibration domain through which membrane-dependent signal prioritization is physiologically modulated.

### 4.2 Controlled carbon dioxide exposure and gaseous modulation of signal competence

A second microenvironmental domain examined during the structural calibration stage concerns the physiological role of carbon dioxide as a regulator of cellular signaling environments. Within biological systems, CO_2_ is not merely a metabolic by-product but a central determinant of acid-base balance, tissue oxygen delivery, and intracellular bioenergetic conditions (Casey et al., 2010; Webb et al., 2011; West, 2012). Variations in local CO_2_ concentration influence extracellular and intracellular pH, modulate membrane electrochemical gradients, and alter the energetic context in which membrane-associated signaling processes occur (Swietach et al., 2007; Swietach et al., 2010; Sinning and Hübner, 2013; Gaspary et al., 2024).

Importantly, CO_2_ levels do not arise solely from host cellular metabolism but reflect the integrated activity of a multigenomic system, including host mitochondrial respiration, microbiota-derived metabolic processes, and broader microenvironmental interactions (Swietach et al., 2010; Gaspary et al., 2024). In this sense, gaseous composition represents a coupled physiological output rather than an isolated physicochemical variable.

One of the primary mechanisms through which CO_2_ influences cellular environments is its role in acid-base regulation via the bicarbonate buffering system. Changes in CO_2_ concentration directly modulate proton availability through carbonic acid dissociation, linking gaseous exchange to intracellular pH and membrane electrochemical states (Casey et al., 2010). Receptor activation, ion channel conductance, and ligand–receptor interactions are sensitive to pH-dependent conformational dynamics; therefore, CO_2_-mediated modulation directly influences the conditions under which extracellular signals achieve signaling competence (Webb et al., 2011; Gaspary et al., 2024).

In addition, CO_2_ regulates tissue oxygen delivery through the Bohr effect, whereby increased CO_2_ and reduced pH facilitate oxygen release from hemoglobin. This mechanism alters mitochondrial respiration and bioenergetic state, which are tightly coupled to redox balance and intracellular signaling pathways (Rich and Maréchal, 2010; Verdin, 2015; Covarrubias et al., 2021). Through these combined effects, CO_2_ modulates not only proton availability but also the energetic conditions required for signal propagation.

These observations extend the formulation introduced in **Section 4.1**. If the effective cellular response depends on *C*_m_, then CO_2_ operates as a physiological regulator of this term by simultaneously modulating proton gradients, membrane electrochemical states, and bioenergetic coupling. Under these conditions, variations in gaseous composition alter the value of *C*_m_, thereby shifting the threshold at which extracellular signals become biologically effective.

However, experimental evidence indicates that modulation of membrane competence alone does not fully determine signaling outcomes. Even when extracellular signals achieve competence at the membrane interface, their biological effect depends on subsequent intracellular processing. This introduces a second constraint: the routing of membrane-competent signals toward intracellular compartments capable of sustaining signaling conversion.

Accordingly, the effective response must account not only for signal availability and membrane competence, but also for routing-dependent propagation, such that:


$$R_{e}ff=S*C_{\text{m}}*W_{\text{r}},$$

Where *W*_r_ represents the intracellular routing weight governing the probability that a competent signal is successfully propagated through signaling pathways capable of generating transcriptional responses. The additional routing term reflects the conceptual distinction between membrane-level competence and successful intracellular propagation, recognizing that membrane-competent signals may still fail to generate regulatory responses if intracellular routing remains inefficient.

Within this extended formulation, CO_2_ influences signaling outcomes through dual mechanisms. First, by modulating *C*_m_ via acid-base and electrochemical effects. Second, by shaping bioenergetic and metabolic conditions that indirectly affect *W*_r_, including endocytic dynamics, adaptor recruitment, and compartmental signaling stability.

Evidence from metabolically active and inflammatory microenvironments further supports this interpretation. Conditions characterized by altered CO_2_ levels, redox imbalance, and metabolic reprogramming reshape signaling landscapes by simultaneously affecting membrane competence and intracellular processing pathways (Nathan and Ding, 2010; Franceschi et al., 2017; Hotamisligil, 2017).

Within the structural calibration logic applied in WP4, the CO_2_ regulatory domain therefore provides a critical test of physiological compatibility. If membrane-dependent signal prioritization constitutes a valid organizing principle, it should remain coherent under conditions in which gaseous composition modulates both the competence and propagation of signaling processes.

From this perspective, carbon dioxide can be understood as a microenvironmental regulator that modulates the physicochemical and energetic landscape within which membrane-dependent signal prioritization operates. Rather than functioning as a classical receptor-mediated signal, CO_2_ shapes the conditions under which extracellular inputs are converted into intracellular regulatory responses.

Consequently, controlled carbon dioxide exposure represents a physiologically plausible domain through which membrane competence and signal routing are jointly modulated under multigenomic microenvironmental conditions.

### 4.3 Proton-structured microenvironments and bioenergetic stabilization mechanisms

A third physiological domain considered during the structural calibration stage concerns the influence of proton-structured microenvironments on cellular bioenergetic regulation. Within biological systems, proton dynamics represent a fundamental determinant of membrane electrochemical states, mitochondrial coupling efficiency, and intracellular pH homeostasis (Mitchell, 1961; Nicholls and Ferguson, 2013; Edgar et al., 2025). Proton gradients directly regulate oxidative phosphorylation and membrane potential across multiple biological membranes; therefore, variations in proton distribution influence the energetic context in which signaling processes occur.

Importantly, proton availability does not arise from isolated cellular processes but reflects the integrated activity of coupled metabolic systems. Host cellular metabolism, microbiota-derived fluxes, inflammatory activity, and local microenvironmental conditions collectively shape proton distribution within tissues (Nicholson et al., 2012; Lynch and Pedersen, 2016). In this sense, proton-structured microenvironments represent emergent physicochemical states that constrain bioenergetic permissivity across coupled biological systems (Gaspary et al., 2026b).

The central role of proton gradients in cellular bioenergetics is well established within the framework of mitochondrial oxidative phosphorylation. Proton transfer across the inner mitochondrial membrane generates the electrochemical gradient required to drive ATP synthesis, linking membrane potential, metabolic flux, and intracellular energetic balance (Rich and Maréchal, 2010). Mitochondrial bioenergetic states influence redox regulation and intracellular signaling networks; therefore, proton dynamics exert indirect but pervasive control over cellular regulatory behavior (Imai and Guarente, 2014; Verdin, 2015; Yoshino et al., 2018; Covarrubias et al., 2021).

Beyond mitochondrial coupling, proton transfer in aqueous environments occurs through coordinated hydrogen-bond networks enabling rapid relay-like mobility. These processes govern electrochemical coupling across membrane interfaces and influence local membrane potential stability. Within cellular systems, such proton-transfer dynamics are tightly integrated with redox homeostasis and stress-response pathways, further linking bioenergetic state to signaling capacity (Wang, 2012; Abrescia et al., 2020).

Microenvironmental metabolic conditions further modulate proton distribution. In metabolically active or pathophysiological environments, including tumor tissues and chronically inflamed systems, altered metabolic flux produces localized shifts in extracellular and intracellular pH. These shifts influence mitochondrial coupling efficiency, cellular adaptation, and immune signaling dynamics, reinforcing the multigenomic nature of the signaling environment (Gaspary et al., 2024; Edgar et al., 2025).

Recent translational analyses suggest that structured proton configurations in aqueous environments, including hydrated complexes such as Eigen (H_9_O_4_^+^) and Zundel (H_5_O_2_^+^), may further influence bioenergetic regulation by modulating proton mobility and electrochemical coupling processes (Edgar et al., 2025). These structures provide an additional layer through which proton dynamics shape the energetic landscape governing cellular signaling.

Within this context, the membrane competence term introduced in Sections 4.1–4.2 can no longer be interpreted as a single scalar variable. Instead, it reflects a composite function integrating multiple structural and energetic determinants of membrane organization. This relationship can be expressed as:


$$C_{\text{m}}=f\left(L,D.E,T.X,O\right),$$

Where membrane competence emerges from the interaction between lipid composition (*L*), microdomain organization (*D*), endocytic routing capacity (*E*), cytoskeletal coupling and membrane tension (*T*), redox and bioenergetic state (*X*), and additional context-dependent modulators (*O*).

This expression should be interpreted as a conceptual state function rather than a quantitative predictive equation, emphasizing that membrane competence emerges from the interaction of multiple physiological determinants rather than from any single isolated variable.

Proton-structured microenvironments exert a distributed influence on membrane competence by simultaneously modulating membrane electrochemical gradients, mitochondrial coupling efficiency, and redox balance. Consequently, signaling competence should be interpreted as a state-dependent property emerging from the integrated organization of membrane structure and cellular energetics rather than as a fixed variable.

These observations further suggest that proton-structured microenvironments function as bioenergetic regulatory layers that both reflect and modulate the integrated activity of multigenomic systems. Rather than acting as classical signaling inputs, proton dynamics redefine the competence landscape through which extracellular signals are evaluated at the membrane interface by shaping receptor clustering stability, membrane potential regulation, and intracellular signaling propagation.

### 4.4 Multigenomic signaling environments and host–exogenous adaptive interfaces

A fourth domain examined during the structural calibration stage concerns the multigenomic nature of the signaling environments within which human physiology operates. Contemporary genomic and metagenomic analyses indicate that the human organism functions as a composite biological system integrating host-derived genetic programs with dynamically interacting microbial, viral, and environmental molecular inputs (Handley, 2016; Sender et al., 2016; Liang and Bushman, 2021).

Within this framework, cellular systems are continuously exposed to heterogeneous extracellular signals originating from multiple biological sources, including host metabolism, microbiota-derived metabolites, viral nucleic acids, extracellular vesicle cargo, and environmentally modulated molecular inputs. These signals coexist within shared molecular environments and interact with common membrane-associated receptor and trafficking systems (Fan and Pedersen, 2021; Wiertsema et al., 2021; Ahmed et al., 2022).

Experimental evidence further demonstrates that these inputs do not operate independently. Microbial metabolites modulate host metabolic and immune pathways, viral components influence immune signaling and microbial dynamics, and extracellular vesicles mediate molecular exchange across biological compartments (He et al., 2018; Cryan et al., 2019; Zhou et al., 2022). As a result, cellular signaling occurs under conditions of continuous molecular coexistence, where multiple inputs compete for interaction with membrane-dependent signaling architectures.

Within such environments, cellular responses no longer reflect the direct integration of all available extracellular signals. Instead, extracellular inputs must first satisfy membrane-dependent conditions of signaling competence and intracellular routing before contributing to regulatory outcomes. Accordingly, effective cellular responses emerge from the selective integration of heterogeneous signals, each differentially weighted by membrane competence and routing efficiency. This relationship can be expressed as:


$$R=\sum \left(S_{\text{i}}*C_{\text{m}}*W_{\text{r}}\right),$$

Where each extracellular input (*S*_i_) contributes to the effective response according to the competence of the membrane interface (*C*_m_) and the efficiency of intracellular routing processes (*W*_r_). The summation represents the concept that cellular responses emerge from the weighted integration of heterogeneous extracellular inputs rather than from isolated ligand-receptor interactions, consistent with the multigenomic framework developed throughout this study.

Within this formulation, the plasma membrane operates as the primary interface through which multigenomic signals are filtered, weighted, and integrated into intracellular regulatory networks. Rather than responding indiscriminately to all inputs, cellular systems generate responses based on the subset of signals that successfully achieve competence and propagation under the prevailing structural and energetic conditions.

This interpretation reinforces membrane-dependent signal prioritization as a physiologically plausible organizing principle of cellular signaling within multigenomic environments. In this context, the plasma membrane can be understood as a biological decision interface through which heterogeneous molecular inputs are selectively translated into cellular regulatory responses.

## 5 Translational implications and experimental research directions

The interpretations presented in this section are derived from structured integrative synthesis and should be understood as components of a hypothesis-derived conceptual framework, as outlined throughout the manuscript, rather than as definitive causal assertions.

Building upon the membrane-dependent signal prioritization architecture established in Sections 4.1–4.4, this section articulates the translational and experimental implications emerging from the formal systems integration conducted in WP5. In this context, the framework generates testable expectations rather than definitive claims, providing a structured basis for investigating how membrane-dependent competence states shape cellular signaling behavior.

Accordingly, the framework predicts that cellular responses to identical extracellular signals may diverge as a function of membrane-dependent competence states, reflecting signaling variability as an emergent property of membrane organization shaped by lipid composition, cytoskeletal coupling, electrochemical state, and intracellular routing dynamics.

### 5.1 The nanobiome as the physicochemical substrate of membrane-dependent signal prioritization

The integrative framework developed in the present study identifies plasma membrane organization as a determinant of signaling competence through mechanisms involving microdomain clustering, routing dynamics, and compartmental signal conversion. While these mechanisms are supported by extensive experimental evidence, their coordinated operation implies the existence of an underlying physicochemical substrate at the nanoscale level in which such processes converge.

Within this context, the concept of the nanobiome is introduced as an integrative interpretive construct describing the nanoscale interfacial environment in which membrane-dependent signal prioritization emerges. The nanobiome is not proposed as a discrete biological entity but as a structured reinterpretation of the plasma membrane interface, including its associated glycocalyx, lipid domains, and adjacent intra- and extracellular microenvironments.

Within this interface, proton dynamics, redox interactions, surface charge distribution, lipid organization, and cytoskeletal coupling collectively define the local conditions under which receptor clustering, signal accessibility, and intracellular routing competence occur. From this perspective, signaling behavior cannot be fully understood as the result of receptor–ligand interactions alone but must be interpreted as an emergent property of the nanoscale environment in which such interactions are embedded.

A key implication of this interpretation is that membrane-dependent signaling operates within a dynamically modulated physicochemical regime. Small variations in interfacial conditions—such as proton concentration, redox balance, or membrane composition—may disproportionately influence signaling outcomes by altering clustering thresholds, routing efficiency, and signal propagation dynamics.

**Figure 4:**
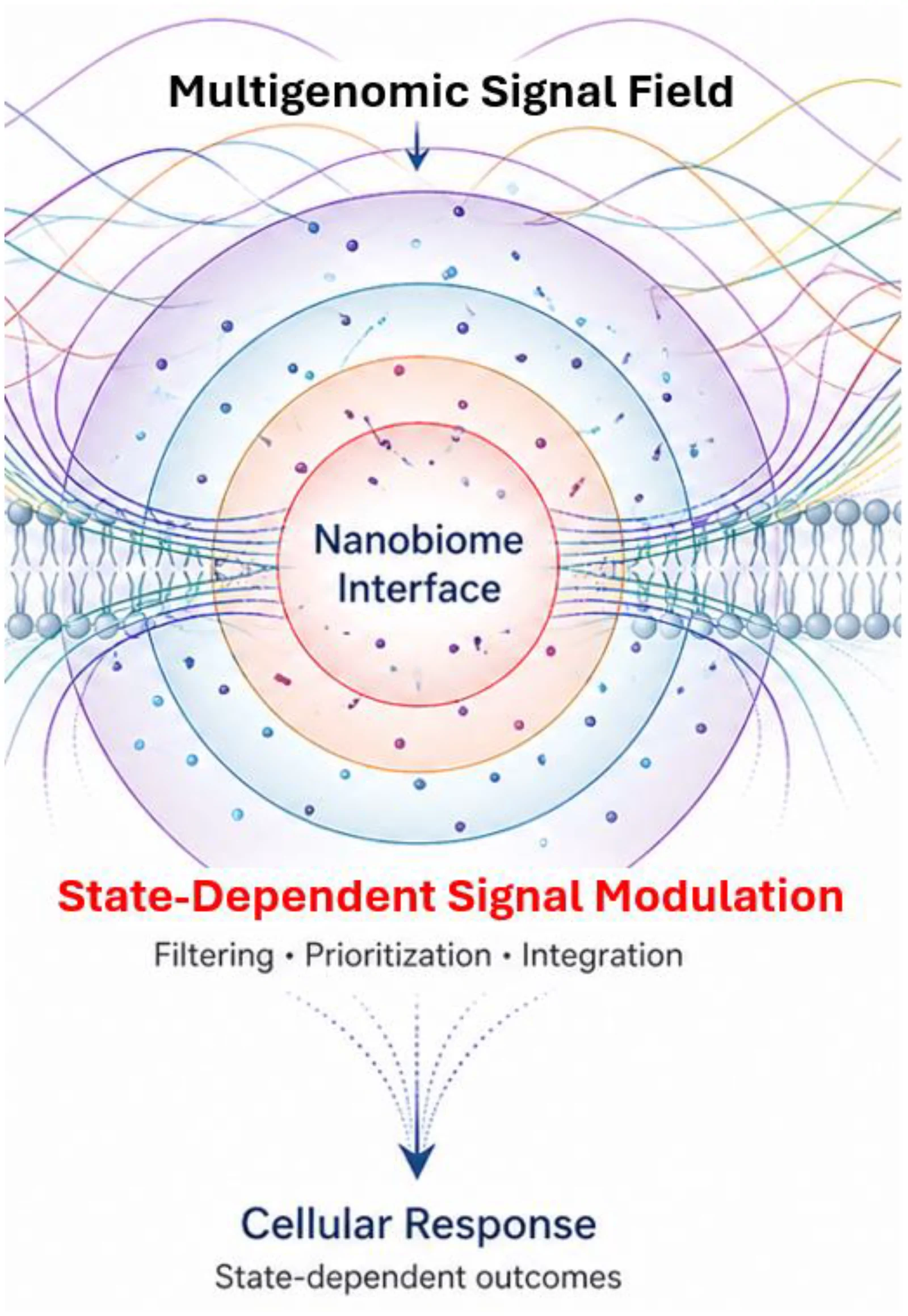
The nanobiome as a nanoscale physicochemical interface for signal modulation.

From this perspective, the nanobiome can be understood as a nanoscale decision field governed by physicochemical constraints, in which extracellular signals are differentially filtered according to their compatibility with local physicochemical conditions (**Figure 4**). Rather than passively receiving molecular inputs, the membrane interface actively conditions which signals are permitted to achieve competence and propagate within intracellular regulatory networks.

This figure illustrates the nanobiome as a nanoscale physicochemical interface at the plasma membrane, where multigenomic and environmental inputs interact with structured electrochemical conditions defined by proton dynamics, redox processes, and surface charge organization. Within this interfacial field, extracellular signals are not directly translated into cellular responses but are modulated through state-dependent constraints that govern their accessibility, clustering potential, and routing competence. Signals that satisfy these conditions contribute to downstream cellular responses, reflecting the selective integration of inputs under the prevailing nanobiome state.

Importantly, this interpretation extends—but does not replace—classical models of membrane signaling. It reorganizes established physicochemical and structural determinants into a coherent interfacial framework, linking membrane organization, bioenergetic modulation, and multigenomic signal integration.

This perspective also provides a mechanistic basis for the state-dependent variability of cellular responses. If signaling competence depends on the physicochemical state of the nanobiome, then identical extracellular signals may produce divergent outcomes depending on local interfacial conditions. Such variability is consistent with experimental observations demonstrating the sensitivity of receptor activation, clustering, and routing dynamics to membrane composition, electrochemical state, and bioenergetic context (Lingwood and Simons, 2010; Kusumi et al., 2012; Bibby et al., 2020; Ruffinatti et al., 2023).

Taken together, the nanobiome concept consolidates the membrane-decisional architecture framework by providing a physically grounded substrate through which signal prioritization is implemented. Within this perspective, membrane-dependent signaling emerges not solely from molecular interactions but from the dynamic configuration of the nanobiome itself.

### 5.2 State-dependent cellular responsiveness and physiological plasticity

A central testable implication of the present framework is that cellular responses to identical extracellular signals may diverge as a function of membrane-dependent competence states, even in the absence of changes in ligand availability or receptor expression. This provides a structured interpretation for the well-recognized observation that similar signaling inputs frequently generate distinct outcomes depending on the physiological state of the cell (Spencer et al., 2009; Altschuler and Wu, 2010). Experimental evidence across diverse systems further indicates that these signaling responses are more closely associated with metabolic context, membrane composition, cytoskeletal organization, and intracellular redox balance than with ligand presence alone (Bibby et al., 2020; Ruffinatti et al., 2023).

Within this framework, such variability reflects state-dependent modulation of membrane organization. Changes in lipid composition, cholesterol distribution, cytoskeletal coupling, and bioenergetic conditions dynamically reshape microdomain structure and intracellular routing behavior, thereby altering clustering thresholds and signaling propagation (Lingwood and Simons, 2010; Kusumi et al., 2012).

Accordingly, modulation of membrane organization is expected to systematically shift signaling outcomes under otherwise identical input conditions. Rather than representing stochastic variability, these differences can be interpreted as structured consequences of changes in membrane competence and routing efficiency.

This interpretation reframes cellular responsiveness as an emergent property of membrane organization rather than a deterministic outcome of receptor–ligand interactions alone. Cells can thus be understood as operating within dynamic signaling states in which membrane-level properties bias the interpretation of extracellular inputs.

Importantly, such state-dependent modulation enables rapid physiological adaptation without requiring immediate transcriptional reprogramming. Variations in metabolic flux, redox balance, or microenvironmental conditions can alter membrane organization and signaling competence, thereby adjusting cellular responsiveness on short timescales.

At a systems level, coordinated shifts in membrane-dependent signaling states across cellular populations are expected to generate aligned physiological responses. Under shared metabolic, inflammatory, or multigenomic conditions, membrane organization may be modulated in a coherent manner across tissues, leading to consistent patterns of cellular responsiveness.

In this sense, physiological behavior can be interpreted as emerging from distributed state-dependent signal prioritization rather than from isolated signaling pathways. Such configurations are consistent with attractor-like dynamics described in systems biology, in which cellular responses converge toward stabilized functional states under persistent conditions (Kauffman, 1993; Kitano, 2002; Noble, 2012).

Importantly, ligand–receptor interactions that do not meet the conditions required for signaling competence are not biologically inert. Within the present framework, such interactions are expected to follow alternative trajectories rather than result in productive signal propagation.

These outcomes may include transient receptor engagement without clustering, receptor internalization followed by recycling or degradation, or subthreshold signaling insufficient to trigger transcriptional activation. In this sense, membrane-dependent constraints do not prevent interaction but modulate its fate within the cellular system.

Accordingly, the absence of downstream signaling should not be interpreted as absence of effect but as a redirection of signaling potential toward attenuated or nonpropagating pathways, reflecting the state-dependent filtering properties of membrane organization.

### 5.3 Distributed signal integration and system-level coordination

A further implication of the proposed framework is that membrane-dependent signal competence, when modulated in a coordinated manner across cellular populations, is expected to generate aligned physiological responses at the organismal level.

Within classical models of physiology, systemic behavior is often interpreted as the downstream integration of signaling cascades occurring within tissues and organs. In contrast, the present framework introduces an upstream layer of regulation in which membrane-dependent signal prioritization operates as a distributed filtering system across large populations of cells.

As membrane organization is influenced by shared metabolic, inflammatory, and microenvironmental conditions, multiple cell types may simultaneously experience shifts in membrane competence and routing efficiency. Under such conditions, signaling responses do not emerge independently but tend to align according to the prevailing physiological context (Levin, 2012; Bibby et al., 2020; Ruffinatti et al., 2023).

This distributed alignment arises because heterogeneous extracellular signals are processed through shared membrane-dependent constraints across tissues. Variations in lipid composition, redox balance, metabolic flux, or multigenomic inputs can therefore produce coordinated shifts in signal prioritization across cellular systems, even in the absence of centralized regulatory control (Bibby et al., 2020; Ruffinatti et al., 2023). In this context, the plasma membrane functions as a shared regulatory substrate through which diverse biological inputs are selectively filtered before entering intracellular signaling pathways.

Consequently, physiological responses traditionally attributed to discrete molecular pathways may instead reflect emergent patterns of distributed signal prioritization across cellular populations. Organism-level behavior can therefore be understood as the integrated outcome of multiple local membrane-dependent decisions shaped by common environmental and metabolic conditions.

**Figure 5:**
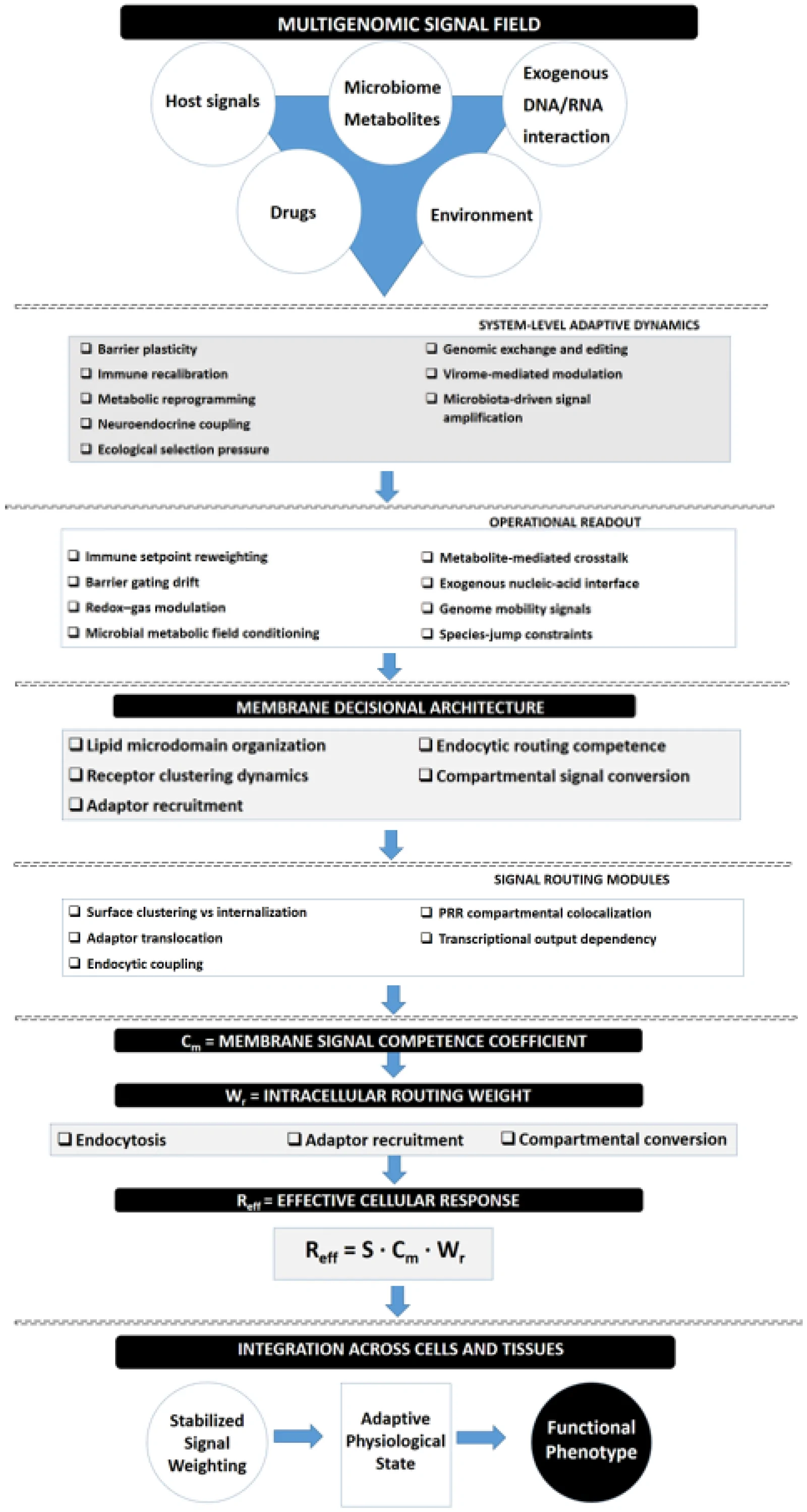
Integrative conceptual architecture linking multigenomic signaling environments to membrane-dependent signal competence and adaptive physiological outcomes.

This interpretation is consistent with systems biology models in which global behavior emerges from the coordinated interaction of local regulatory units. Under persistent conditions, such distributed processes may converge toward coherent and potentially stabilized physiological configurations without requiring explicit hierarchical control (Kauffman, 1993; Kitano, 2002; Noble, 2012). The integrative conceptual architecture emerging from this framework is summarized in **Figure 5**.

The upper layer represents the heterogeneous extracellular signaling environment in which human cells operate, including host-derived mediators, microbiota-associated metabolites, viral nucleic acids, extracellular vesicle cargo, pharmacological agents, and environmentally derived molecular inputs. Together, these signals constitute the multigenomic pool of extracellular stimuli available to cellular systems.

The intermediate layers illustrate system-level adaptive dynamics through which extracellular signals influence host physiology, including barrier plasticity, immune recalibration, metabolic reprogramming, neuroendocrine coupling, ecological selection pressures, and virome-mediated modulation. Within this regulatory landscape, extracellular inputs interact with host cellular systems through complex molecular exchanges and microenvironmental modulation.

The central structural layer represents the Membrane Decisional Architecture identified through mechanistic synthesis in WP2 and WP3. Membrane lipid microdomain organization, receptor clustering dynamics, adaptor recruitment, and endocytic routing competence collectively determine whether extracellular molecular inputs achieve signaling competence at the membrane interface.

The subsequent layers represent intracellular signal routing modules, including receptor internalization dynamics, adaptor coupling, compartmental signal conversion, and transcriptional pathway engagement. These processes determine whether membrane-competent signals are successfully propagated through intracellular regulatory networks.

At the organismal level, the integration of effective cellular responses across tissues gives rise to stabilized signal-weighting patterns that shape adaptive physiological states and contribute to the functional phenotype of the organism.

### 5.4 A starting point for plasma membrane nanobiome monitoring

A further experimentally tractable implication emerging from the formal systems integration conducted in WP5 concerns the role of interfacial electrochemical conditions as modulators of membrane signal competence. If *C*_m_ emerges from electrochemical and physicochemical determinants, then both proton dynamics and membrane electrical potential constitute primary variables governing signaling permissivity at the membrane interface (Mitchell, 1961; Nicholls and Ferguson, 2013).

From an electrochemical standpoint, the behavior of molecules at the membrane interface cannot be described solely in terms of concentration gradients. Instead, it is defined by the coupled influence of local proton activity and the electrical potential difference across the plasma membrane (Δφ), which together determine the distribution and movement of charged and partially charged species. Quantitative models of transmembrane transport demonstrate that passive fluxes depend jointly on pH and Δφ, reflecting the coupled electrochemical driving forces governing molecular behavior across lipid bilayers (Mitchell, 1961; Nicholls and Ferguson, 2013; Gąbka et al., 2021). This coupling has been explicitly modeled in the context of weak acid transport across membranes, where both proton gradients and membrane potential determine intracellular accumulation dynamics (Noble, 2012).

Under these conditions, the identification of an experimentally tractable reference condition requires selecting a regime in which variations in proton activity translate into measurable changes in interfacial electrochemical organization. Under physiologically compatible aqueous conditions, this regime is expected to lie within the near-neutral range, operationally approximated here as a near-neutral transition interval (~ 6.5–6.9). This range is not proposed as an optimal physiological state but rather as a transition interval in which incremental changes in proton concentration produce disproportionate effects on molecular protonation states and, consequently, on surface charge distribution.

As protonation directly modulates the charge of membrane-associated molecules, variations in perimembrane pH are expected to alter the electrostatic boundary conditions at the membrane interface. These changes are inherently reflected in the interfacial electrostatic potential, commonly described as zeta potential, which provides an experimentally accessible approximation of interfacial charge organization. In this sense, pH and Δφ jointly define the electrochemical landscape of the membrane, while zeta potential represents its measurable projection.

This relationship becomes particularly relevant in membrane-associated microenvironments, where heterogeneous dielectric properties, surface charge distributions, and nanoscale confinement can shift effective protonation equilibria and amplify the impact of subtle variations in proton activity. Under such conditions, small deviations in perimembrane pH within the defined transition range may induce measurable changes in zeta potential, thereby modulating electrostatic repulsion, lateral mobility, receptor clustering, and endocytic routing dynamics. These mechanisms are consistent with evidence demonstrating that pH variations influence receptor conformation, ion channel activity, and ligand–receptor interaction dynamics (Casey et al., 2010; Wang, 2012), as well as membrane organization and trafficking processes (Lingwood and Simons, 2010; Kusumi et al., 2012).

Importantly, this interpretation does not posit a specific optimal pH value but rather defines an electrochemically sensitive regime in which protonation-dependent modulation of interfacial charge and field effects exerts a disproportionate influence on membrane signal competence. Accordingly, controlled perturbations within this range are expected to introduce measurable constraints on receptor conformation, clustering thresholds, surface charge distribution, and intracellular routing efficiency.

Experimental designs may therefore investigate how controlled modulation of localized perimembrane pH within this transition range (~ 6.5–6.9), in conjunction with intrinsic membrane electrical potential, alters receptor clustering, endocytic routing, and transcriptional responses under otherwise identical extracellular conditions. pH cannot be assigned to the membrane nanobiome as a bulk property; therefore, this variable should be operationalized as membrane-proximal proton activity or interfacial pH, measured using surface-targeted pH-sensitive probes, membrane-anchored fluorescent sensors, or related high-resolution approaches. In this context, monitoring pH-dependent shifts in zeta potential provides a direct strategy for probing variations in *C*_m_, offering a practical entry point for assessing nanobiome-dependent changes in signaling permissivity.

This interpretation also aligns with evidence indicating that gene expression reflects state-dependent cellular organization rather than isolated molecular triggers. Within this framework, epigenetic configurations can be understood as downstream readouts of membrane-mediated state organization in which variations in proton dynamics, electrical potential, and signaling propagation influence chromatin accessibility and transcriptional activity (Noble, 2012). Accordingly, cellular responses—and their associated gene expression profiles—emerge from the subset of signals that successfully achieve competence within the membrane interface.

**Figure 6:**
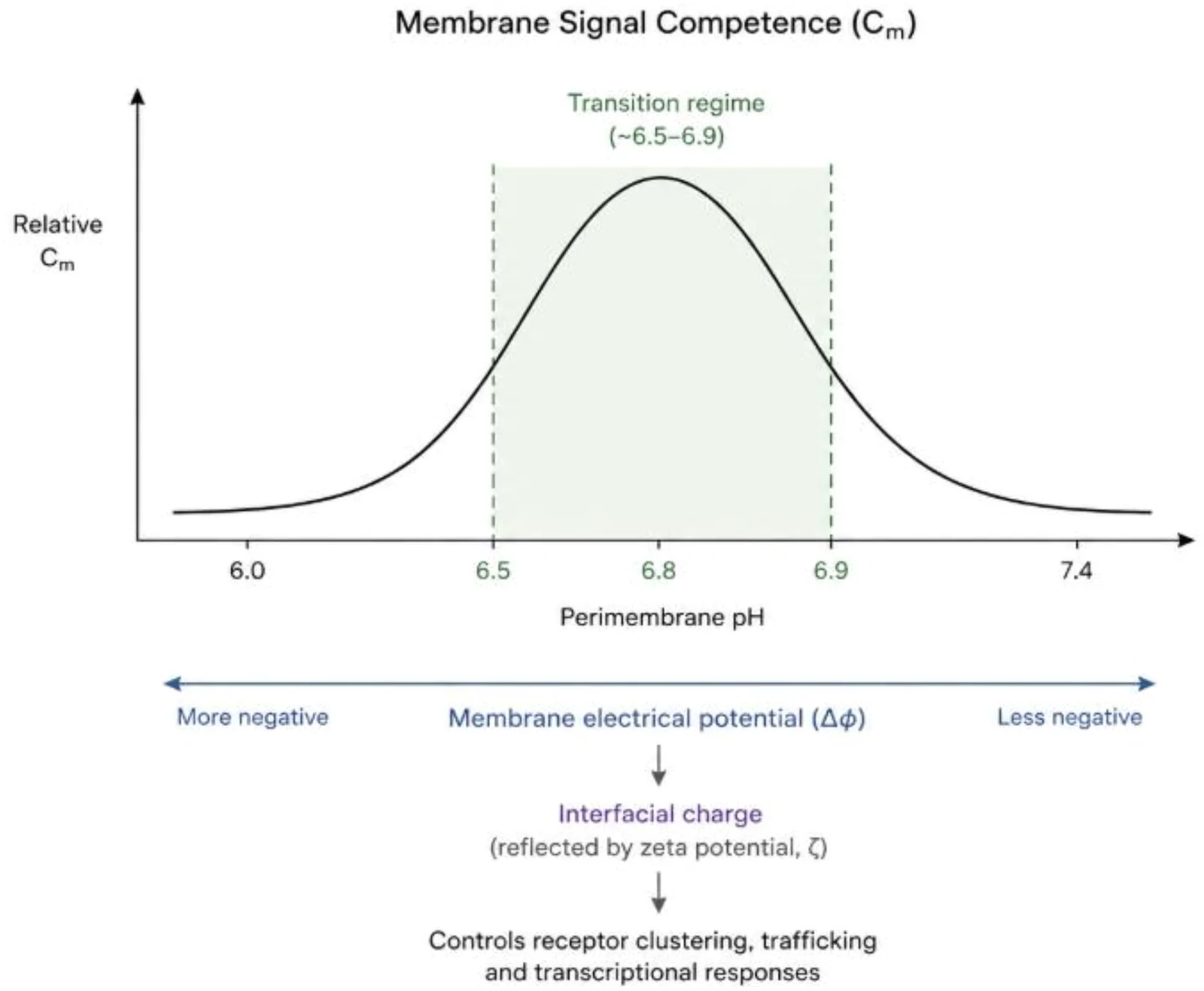
Electrochemical transition regime governing membrane signal competence.

As illustrated in **Figure 6**, *C*_m_ can be represented as an electrochemical state-space, in which a near-neutral transition regime (~ 6.5–6.9) defines a region of heightened electrochemical sensitivity where small perturbations in proton activity and membrane potential produce amplified effects on interfacial organization.

*C*_m_ emerges from the coupled influence of perimembrane proton activity (pH) and Δφ, which jointly determine interfacial electrostatic conditions. Within a near-neutral transition regime (~ 6.5–6.9), small variations in proton concentration—modulated by the intrinsic electrical potential—produce disproportionate changes in surface charge organization, experimentally reflected by zeta potential (ζ). These interfacial conditions regulate receptor clustering, membrane trafficking, and downstream transcriptional responses, providing an operational framework for probing nanobiome-dependent variations in signaling permissivity.

These experimentally grounded implications provide the basis for a broader conceptual interpretation of membrane-dependent signaling within physiological systems, discussed in the following section.

## 6 Discussion

The present study advances a hypothesis-derived integrative framework grounded in the structured synthesis of experimentally established mechanisms. Accordingly, the interpretations discussed below should be understood as explanatory constructs intended to organize and extend existing knowledge rather than as definitive causal claims. Within this perspective, convergent evidence from membrane biology, receptor trafficking, and cellular signaling is reorganized into a unified physiological interpretation in which plasma membrane organization functions as a structural interface governing signaling competence (Lingwood and Simons, 2010; Kusumi et al., 2012; Chen et al., 2023; Armstrong et al., 2025), without proposing new molecular mechanisms but instead reframing how established processes are integrated within cellular systems.

A central implication of this synthesis is that cellular signaling cannot be fully explained by ligand availability or receptor expression alone. Rather, signaling outcomes emerge from membrane-dependent conditions that determine whether extracellular inputs acquire functional competence and how they are propagated within intracellular networks (Spencer et al., 2009; Altschuler and Wu, 2010; Bibby et al., 2020; Ruffinatti et al., 2023). This perspective therefore challenges ligand-centric models by introducing an upstream organizational layer that constrains signal integration before canonical signaling cascades are engaged.

### 6.1 Reframing cellular signaling: from input- to competence-driven systems

Traditional models of cellular signaling implicitly assume that biological responses are primarily determined by the identity and concentration of extracellular inputs. While this assumption has proven useful, it remains insufficient to explain why similar signals frequently produce divergent outcomes across different physiological contexts (Spencer et al., 2009; Altschuler and Wu, 2010). The present framework suggests that such variability reflects differences in membrane-dependent signal competence rather than inconsistencies in signaling pathways, proposing a competence-driven model in which extracellular inputs must first satisfy structural and organizational conditions at the membrane interface before generating effective cellular responses. Under this interpretation, the plasma membrane functions not as a passive boundary but as a regulatory interface governing the conditions under which signals become biologically effective (Lingwood and Simons, 2010; Kusumi et al., 2012).

### 6.2 Integration with classical physiology

Importantly, this framework does not contradict classical models of physiology but extends them by integrating established concepts—including membrane microdomains, receptor clustering, endocytic trafficking, and compartmental signal conversion—into a coherent physiological interpretation centered on signaling competence (Lingwood and Simons, 2010; Watanabe et al., 2011; Kusumi et al., 2012; Chen et al., 2023).

Within this interpretation, the plasma membrane can be understood as a generalized gating system, analogous to well-characterized gating mechanisms in ion channels and excitable tissues (Amarillo et al., 2008; Han et al., 2018; Kuo et al., 2020). However, rather than regulating ion flux alone, membrane organization governs signaling competence across diverse classes of extracellular inputs—including ligands, nucleic acids, metabolites, and environmental molecular signals—providing a unified explanation for the context-dependent variability of signaling observed across multiple biological systems (Spencer et al., 2009; Altschuler and Wu, 2010; Bibby et al., 2020; Ruffinatti et al., 2023).

### 6.3 Relationship to existing membrane signaling models

The conceptual framework proposed in the present study is intended to complement rather than replace existing models of membrane biology. Its primary contribution does not lie in introducing new molecular mechanisms but in providing a higher-order physiological architecture through which experimentally established mechanisms can be interpreted as components of a unified membrane-dependent signaling framework.

The lipid raft hypothesis established membrane microdomains as fundamental organizing principles of the plasma membrane, demonstrating that cholesterol- and sphingolipid-enriched nanodomains regulate receptor localization, molecular clustering, and signaling efficiency. Subsequent work further refined this view by describing the plasma membrane as a dynamic and continuously reorganized signaling platform rather than a homogeneous fluid surface (Simons and Ikonen, 1997; Brown and London, 2000; Pike, 2006; Lingwood and Simons, 2010; Kusumi et al., 2012).

Complementary advances in endosomal and membrane-dependent signaling demonstrated that productive signaling frequently depends not only on receptor engagement but also on intracellular trafficking, adaptor recruitment, compartmental signal conversion, and membrane-associated retention mechanisms (Watanabe et al., 2011; Chen et al., 2023; Armstrong et al., 2025). These studies established that signaling competence emerges through coordinated membrane organization and intracellular routing rather than through ligand–receptor interaction alone.

The present framework incorporates these experimentally established mechanisms while extending their interpretation to the systems level. Rather than treating membrane organization, receptor clustering, intracellular routing, and physicochemical modulation as separate explanatory domains, membrane-decisional architecture interprets them as coordinated components of a single membrane-dependent competence system operating under heterogeneous multigenomic signaling environments. Within this interpretation, membrane organization functions not merely as a structural scaffold or trafficking platform but as the upstream interface through which extracellular inputs are selectively prioritized before entering intracellular regulatory pathways.

The principal contribution of the proposed framework lies not in introducing new molecular mechanisms but in organizing established mechanisms into a coherent and self-organizing physiological architecture of membrane-dependent signaling competence. The framework does not propose new membrane structures, signaling pathways, or molecular entities. Instead, it integrates established membrane biology, trafficking dynamics, bioenergetic modulation, and multigenomic physiology into a unified explanatory framework centered on signaling competence. In this sense, the proposed architecture organizes existing evidence into a coherent system-level model of membrane-dependent signal prioritization.

### 6.4 State-dependent responsiveness as a systems property

The framework further supports the view that cellular responsiveness is inherently state-dependent. Rather than reflecting stochastic variability, differences in signaling outcomes across cells and conditions can be understood as structured consequences of variations in membrane organization, metabolic context, and bioenergetic state (Spencer et al., 2009; Altschuler and Wu, 2010; Ruffinatti et al., 2023).

Importantly, the present framework operates at two complementary levels. Membrane-dependent signal prioritization represents a generalizable organizational principle of cellular systems, grounded in conserved mechanisms such as membrane microdomain organization, receptor clustering, and intracellular routing. Its operational manifestation, however, remains inherently context-dependent, as variations in membrane composition, receptor repertoire, cytoskeletal organization, and bioenergetic state modulate membrane competence and routing efficiency across distinct cell types. Consequently, while the underlying principles of membrane-decisional architecture may be broadly applicable across biological systems, signaling outcomes are expected to vary across epithelial, immune, neuronal, metabolic, and other physiological contexts, consistent with system-level interpretations in which conserved structural principles generate heterogeneous functional outcomes depending on local system states (Kauffman, 1993; Kitano, 2002; Noble, 2012).

Within this perspective, cells operate within dynamic signaling states in which membrane properties bias the interpretation of extracellular inputs. These states are continuously modulated by metabolic flux, redox balance, and microenvironmental conditions, allowing rapid adaptation without requiring immediate genomic reprogramming (Casey et al., 2010; Verdin, 2015; Covarrubias et al., 2021).

At a systems level, coordinated shifts in these membrane-dependent states across cellular populations may lead to aligned physiological responses. Such distributed alignment suggests that organism-level behavior can emerge from the synchronization of local signal prioritization processes occurring at the membrane interface, rather than from centralized regulatory control (Kauffman, 1993; Kitano, 2002; Levin, 2012; Noble, 2012).

### 6.5 Toward a system-level interpretation of physiological states

A key implication of this interpretation is that physiological behavior may be understood as emerging from structured patterns of signal prioritization across cellular systems. Under persistent environmental and metabolic conditions, membrane-dependent signaling states may converge toward stabilized configurations of cellular activity (Kauffman, 1993; Kitano, 2002; Noble, 2012).

From a conventional biomedical perspective, such configurations are often interpreted as pathophysiological states. The present framework does not challenge the clinical reality of disease. Rather, it suggests that certain conditions may be more appropriately understood as stabilized physiological configurations emerging from sustained patterns of membrane-dependent signal prioritization under specific environmental and metabolic constraints (Medzhitov, 2008; Netea et al., 2016; Netea et al., 2020).

Understanding how these configurations arise and stabilize may therefore provide a basis for interpreting physiological variability, resilience, and dysfunction [20.22,24].

### 6.6 Predictive implications: attractor-based interpretation of membrane-dependent signaling states

The framework proposed in this study also allows for a system-level interpretation grounded in dynamical systems theory. Membrane-dependent signal competence can be understood as a local determinant shaping the trajectory of cellular states within a multidimensional physiological state space.

Within this perspective, cellular signaling behavior evolves within structured regions of stability—commonly described as attractor states—emerging from the integration of metabolic, redox, neuroendocrine, and signaling dynamics (Kauffman, 1993; Kitano, 2002; Alon, 2007; Noble, 2012).

The present framework suggests that membrane organization contributes to defining both the accessibility and stability of these attractor states by modulating signal competence thresholds. Under this interpretation, identical extracellular inputs may lead to distinct cellular trajectories depending on the underlying membrane competence state, providing a mechanistic basis for state-dependent responsiveness (Spencer et al., 2009; Altschuler and Wu, 2010; Ruffinatti et al., 2023).

At the systems level, coordinated modulation of membrane-dependent signaling competence across cellular populations may influence the emergence and stabilization of organism-level physiological configurations. In this sense, membrane-level signal prioritization can be interpreted as a contributing factor to the formation of dynamically stabilized regulatory regimes under heterogeneous signaling conditions (Levin, 2012; Noble, 2012).

This attractor-based interpretation does not introduce new mechanisms but provides a system-level extension of the proposed framework, linking membrane-dependent signaling competence to the broader dynamics of physiological state transitions. Accordingly, attractor-state dynamics should be interpreted as a system-level conceptual extension of the proposed architecture rather than as an experimentally established property of membrane-dependent signaling itself.

### 6.7 Conceptual boundaries and limitations

The framework proposed in this study is conceptual and synthesis-driven in scope. It does not introduce new molecular entities nor establish direct causal relationships between specific extracellular signals and defined physiological outcomes. Instead, it provides an organizational interpretation that integrates established mechanisms into a coherent physiological model.

A central limitation of this approach lies in its hypothesis-generating nature. The membrane-decisional architecture is proposed as a conceptual variable derived from structured integrative synthesis rather than from direct experimental isolation. While the underlying phenomena—such as receptor clustering, membrane-dependent routing, and context-dependent signaling variability—are extensively documented, their formalization into a unified explanatory construct remains to be empirically validated as an independent variable. Accordingly, the framework would require revision if future experimental evidence consistently demonstrated that membrane organization, routing dynamics, and physicochemical membrane states do not systematically contribute to signaling competence beyond what can be explained by ligand availability and receptor abundance alone.

This limitation reflects a broader gap within current signaling biology. Although context-dependent variability in cellular responses is widely recognized, there is no widely adopted unifying variable that prospectively links membrane structure, signal accessibility, and intracellular propagation. As a result, signaling variability is often explained retrospectively through multiple interacting factors, without a structured framework that defines the conditions under which signals become competent. The present framework attempts to address this gap; however, its integrative nature also makes it inherently challenging to validate using conventional reductionist methodologies.

This introduces an inherent methodological tension. The framework operates at a level intended to generate integrative interpretations and guide future investigation, yet it may be evaluated under criteria typically applied to validated mechanistic models. Under such conditions, broader conceptual statements may be perceived as insufficiently constrained, whereas restricting the framework to only experimentally validated claims would reduce it to a descriptive account of already known processes, thereby limiting its integrative contribution.

In addition, the framework explicitly seeks to reduce a recurrent source of interpretative circularity in signaling biology, in which outcomes are attributed to “context” without clearly defining the structural and physicochemical conditions that constitute that context. By introducing membrane-dependent signal competence as an organizing variable, the present work aims to make these conditions explicit. However, this effort remains limited by the current absence of experimental systems designed to isolate and manipulate this variable as a discrete entity.

Additional conceptual boundaries should be emphasized. The term “decisional” is used strictly to describe emergent outcomes of structural and trafficking constraints, without implying intentionality or cognitive processes. Similarly, within the present framework, multigenomic signaling should not be interpreted as implying autonomous control by exogenous systems but as reflecting the integration of heterogeneous inputs within a host-regulated membrane interface.

Finally, membrane-dependent signal prioritization should be understood as one layer within a broader hierarchy of physiological regulation. Downstream processes—including transcriptional control, metabolic adaptation, and tissue-specific signaling networks—remain essential components of biological function. The framework therefore does not replace existing models but proposes an additional organizational layer through which signaling competence is regulated.

Recognizing these limitations is essential for situating the present framework within its appropriate scientific context: as a hypothesis-derived, integrative construct grounded in established mechanisms and intended to guide experimental investigation rather than to assert a definitive causal structure.

### 6.8 Conceptual synthesis and implications

Taken together, the framework proposed in this study suggests a shift in how cellular signaling and physiological regulation are interpreted. Rather than viewing biological responses as direct consequences of molecular inputs, the present work supports the interpretation that signaling outcomes emerge from membrane-dependent conditions that define signal competence and propagation.

Within this perspective, physiological states can be understood as structured outcomes of distributed signal prioritization processes occurring at the membrane interface. Under persistent conditions, these processes may stabilize into coherent configurations that shape organism-level behavior.

This interpretation does not replace existing models of physiology but reframes them within a broader organizational context in which membrane architecture acts as an upstream determinant of signaling variability and integration. In doing so, the framework provides a conceptual bridge between mechanistic observations and system-level behavior, offering a foundation for future experimental and theoretical investigation into how biological systems maintain stability while remaining adaptable to complex and heterogeneous environments.

## 7 Conclusion

The present work advances the interpretation that plasma membrane organization constitutes a previously underappreciated layer governing the transition from signal presence to signaling competence. By synthesizing experimental findings from membrane biology, receptor trafficking, and cellular signaling, this study proposes membrane-decisional architecture as a framework for understanding how cells selectively interpret heterogeneous extracellular inputs.

Within this framework, membrane microdomains and associated trafficking architectures regulate a sequence of conditional transitions through which signals acquire biological effectiveness. Receptor clustering establishes structural competence, membrane-dependent routing defines intracellular trajectories, and compartmental conversion enables transcriptional activation. Together, these processes implement a membrane-governed signal prioritization system that determines which extracellular inputs acquire regulatory relevance within cellular signaling networks.

This interpretation provides a physiologically grounded explanation for longstanding observations in cellular biology, including the dissociation between ligand binding and functional signaling responses, the context-dependent nature of cellular responsiveness, and the selective integration of heterogeneous molecular inputs within complex biological environments.

Beyond its mechanistic implications, the framework suggests that cellular regulatory behavior may emerge from structured patterns of signal prioritization occurring at the membrane interface. As signaling networks respond to the subset of inputs that successfully achieve competence, sustained membrane-level conditions may contribute to the stabilization of coherent physiological configurations within human systems.

Importantly, membrane-decisional architecture does not replace established signaling models but extends them by introducing an upstream organizational layer through which signals acquire competence before entering canonical signaling cascades. In this sense, the plasma membrane functions as a dynamic integrative interface that filters, prioritizes, and translates extracellular inputs into coordinated cellular responses.

Rather than reflecting the totality of signals present in the extracellular environment, cellular signaling reflects the subset that becomes competent under membrane-dependent conditions. Recognizing this interface as a determinant of signaling competence provides a unifying physiological perspective on how cells maintain responsiveness and stability within multigenomic environments.

This perspective opens a path for experimentally defining membrane competence thresholds as measurable determinants of physiological variability across multigenomic environments. Future investigations may therefore explore how membrane-level structural dynamics define measurable competence thresholds across physiological contexts and how these thresholds govern the emergence, stabilization, and transition of adaptive physiological states.
